# *TLR1*, *2*, *4*, *6* and *9* Variants Associated with Tuberculosis Susceptibility: A Systematic Review and Meta-Analysis

**DOI:** 10.1371/journal.pone.0139711

**Published:** 2015-10-02

**Authors:** Haiko Schurz, Michelle Daya, Marlo Möller, Eileen G. Hoal, Muneeb Salie

**Affiliations:** SA MRC Centre for Tuberculosis Research and the DST/NRF Centre of Excellence for Biomedical Tuberculosis Research, Division of Molecular Biology and Human Genetics, Faculty of Medicine and Health Sciences, Stellenbosch University, Tygerberg, South Africa; University of Birmingham, UNITED KINGDOM

## Abstract

**Background:**

Studies investigating the influence of toll-like receptor (*TLR*) polymorphisms and tuberculosis susceptibility have yielded varying and often contradictory results in different ethnic groups. A meta-analysis was conducted to investigate the relationship between *TLR* variants and susceptibility to tuberculosis, both across and within specific ethnic groups.

**Methods:**

An extensive database search was performed for studies investigating the relationship between TLR and tuberculosis (TB) susceptibility. Data was subsequently extracted from included studies and statistically analysed.

**Results:**

32 articles involving 18907 individuals were included in this meta-analysis, and data was extracted for 14 *TLR* polymorphisms. Various genetic models were employed. An increased risk of TB was found for individuals with the *TLR2* rs3804100 CC and the *TLR9* rs352139 GA and GG genotypes, while decreased risk was identified for those with the AG genotype of *TLR1* rs4833095. The T allele of *TLR6* rs5743810 conferred protection across all ethnic groups. *TLR2* rs5743708 subgroup analysis identified the A allele to increase susceptibility to TB in the Asian ethnic group, while conferring protection in the Hispanic group. The T allele of *TLR4* rs4986791 was also found to increase the risk of TB in the Asian subgroup. All other *TLR* gene variants investigated were not found to be associated with TB in this meta-analysis.

**Discussion:**

Although general associations were identified, most *TLR* variants showed no significant association with TB, indicating that additional studies investigating a wider range of pattern recognition receptors is required to gain a better understanding of this complex disease.

## Introduction

Tuberculosis (TB), caused by *Mycobacterium tuberculosis* (*M*. *tuberculosis*), is the leading cause of death attributable to a single infectious agent worldwide [1]. The host innate immune response is the first line of defence against invading pathogens and is vital for the initial defence against *M*. *tuberculosis* and activation of the adaptive immune response [2]. This primary immune response is induced by binding of conserved structures in the cell wall or genetic components of the invading pathogen, termed pathogen associated molecular patterns (PAMPs), to host pattern recognition receptors (PRRs) [3]. These PRRs include the toll-like receptors (TLRs), C-type lectin receptors (CLRs), nucleotide-binding oligomerization domain (NOD)-like receptors (NLRs) and the RIG-like receptors (RLRs) [3]. For the purpose of this meta-analysis we focussed on the TLRs as they are the most extensively studied family of PRRs. PRRs, which recognise the PAMPs, are mostly germline encoded receptors expressed on immune cells, including macrophages and dendritic cells [4] and are expressed either on the extracellular cell surface (TLR1, 2, 4, 5, 6 and CLRs) or intracellularly in the cytosol or on endosomal membranes (TLR3, 7, 8, 9, NLRs and RLRs).

As the PRR encoding genes play an important role in host immunity, variants in these genes could lead to structural and functional changes in these receptors causing an altered immune response, and influence TB disease progression [3].

TLR2 and 4 are the most studied TLRs with regards to TB disease. TLR2 forms heterodimers with either TLR1 or TLR6, resulting in the recognition of a wide range of mycobacterial PAMPs, including tri and diacyl lipopeptides [5] and peptidoglycan [6]. Given that TLR2 forms heterodimers it is clear that defects in this gene could influence ligand recognition of multiple receptors, which could affect the host’s innate immune response and thus alter susceptibility to TB disease. Multiple studies on various *TLR2* SNPs have been conducted, often with varying and even contradictory results in different ethnic groups. The A allele of the *TLR2* rs11938228 polymorphism has been associated with TB disease (allelic and recessive model) in European and Asian populations, but not African [7] or Hispanic [8] populations. Another study in an Asian [9] cohort found no association. Similar conflicting results have been found for *TLR4* polymorphisms. TLR4 recognises mycobacterial lipopolysaccharides (LPS) and can trigger one of two innate immune response pathways, the MyD88 dependant or independent pathway. Impairments in this PRRs signalling capability can greatly influence TB disease susceptibility [10]. *TLR4* rs4986790 and rs4986791 are two of the most extensively investigated and were shown to be associated with TB susceptibility, for the allelic and heterozygous model, in one Asian population [11] but not in a second Asian cohort [9]. In an African population the rs4986791 polymorphism was absent, while the rs4986790 had no influence on disease susceptibility [12]. While some of this variation in results can be attributed to small sample sizes it is clear that the genetic make-up of diverse ethnic groups may also play a major role in TB disease susceptibility.

Meta-analysis enables us to systematically review the results of previous studies to derive a relevant, objective and unbiased conclusion by taking into account the totality of evidence on a specific subject [13]. By considering and aggregating as much data as possible on a specific topic using statistical measures, the sample size and thus the power to find an association is increased [14]. Here we performed a meta-analysis on the most commonly investigated *TLR1*, *2*, *4*, *6*, *8*, and *9* SNPs, to assess their association with TB susceptibility both across and within different ethnicities. We show that most of the commonly investigated SNPs have no association with TB disease susceptibility across ethnic groups. Subgroup analysis was possible for eight SNPs and two of these were significant in our analysis. Four SNPs (*TLR1* rs4833095, *TLR2* rs3804100, *TLR6* rs5743810 and *TLR9* rs352139) were associated with TB susceptibility across ethnic groups, while subgroup analysis on *TLR2* rs5743708 and *TLR4* rs4986791 showed significant association in the Asian and Hispanic ethnic groups. Further investigation to validate these findings will be required as more studies from various ethnic groups become available.

## Materials and Methods

### Publication search

A systematic search of articles relating to variants in *TLR* genes and susceptibility to TB was conducted, by two researchers (HS and MS), using the PubMed, Medline and EMBASE databases, including studies up to 31 May 2015. The search strategy was based on various combinations of the following terms: “TLR”, “toll-like” or “toll like” in combination with “tuberculosis”, “TB”, “M.tb” or “mycobacteria” and in conjunction with “genotype”, “allele”, “polymorphism” or “variant”. Furthermore, the reference lists of the publications identified were searched for further relevant studies. If data was missing the corresponding author of the study was contacted via e-mail to obtain missing data where possible.

### Inclusion and exclusion criteria

The following criteria were required for inclusion of studies: (1) case-control study; (2) evaluation of *TLR* variants and TB or pulmonary TB (pTB) susceptibility; (3) genotype frequencies for both cases and controls; (4) Newcastle Ottawa Scale (NOS) quality score of ≥6 [15]. Studies were excluded if they: (1) did not deal with humans, TLR or *M*. *tuberculosis*; (2) review articles or previous meta-analyses; (3) insufficient or duplicate data; (4) not in English.

### Data extraction

For all eligible studies the following data was extracted from the original publications: title, first author and year of publication, ethnicity of study population, number of cases and controls, and genotype frequencies for cases and controls. The data extraction was done independently by HS and MS and then compared to detect any discrepancies.

### Statistical analysis

Analysis of the extracted data was performed using the freely available R programming environment v3.1.2 (http://www.r-project.org/). Hardy-Weinberg Equilibrium (HWE) was calculated for the control group of each study using the Chi-square test in the *HardyWeinberg* package v1.4.1 (http://cran.r-project.org/). Odds ratios (OR) and 95% confidence intervals (CI) for each study and the pooled result was calculated to assess the association between *TLR* variants and TB susceptibility. The allelic model (2 vs. 1), homozygote comparison (22 vs. 11), heterozygote comparison (12 vs. 11), dominant model (22 + 12 vs. 11) and recessive model (22 vs. 12 + 11) was analysed for each SNP (if data from three or more studies were available) using the *metafor* package v1.6–0 (http://cran.r-project.org/). Analysis was also performed by ethnicity (Asian, African, European, and Hispanic) if at least three articles for a specific ethnicity was available. The Chi-squared based Q statistic and *I* ^2^ test was used to assess the heterogeneity between included studies [16]. For a heterogeneity result of *p*-value > 0.1 the fixed effects (FE) model (inverse-variance method) was implemented and for *p*-value ≤ 0.1 the random effects (RE) model (restricted maximum likelihood estimator) was used to calculate pooled OR and CI values [17]. For each statistical model a *p*-value <0.05 was considered significant. Genotypes that displayed deviations from HWE or studies that had a low NOS score were excluded from this meta-analysis. Due to these exclusions sensitivity analysis was not strictly necessary, but was performed to ensure stability of the meta-analysis, based on the *I* ^2^ statistic. Confounding factors (gender and environmental interactions) were not included in the analysis as this information was not available for all included studies. Finally, publication bias was assessed using Egger’s weighted regression test with inverse sample size estimator, as this gives a better estimate of bias than the more commonly used standard error estimator if the number of studies included are limited [18]. A *p*-value < 0.05 was taken to indicate the presence of publication bias, but was only considered valid if more than five studies were included. Any bias was corrected for using the Duval and Tweedie trim and fill method [19].

## Results

### Study characteristics

**Fig 1:**
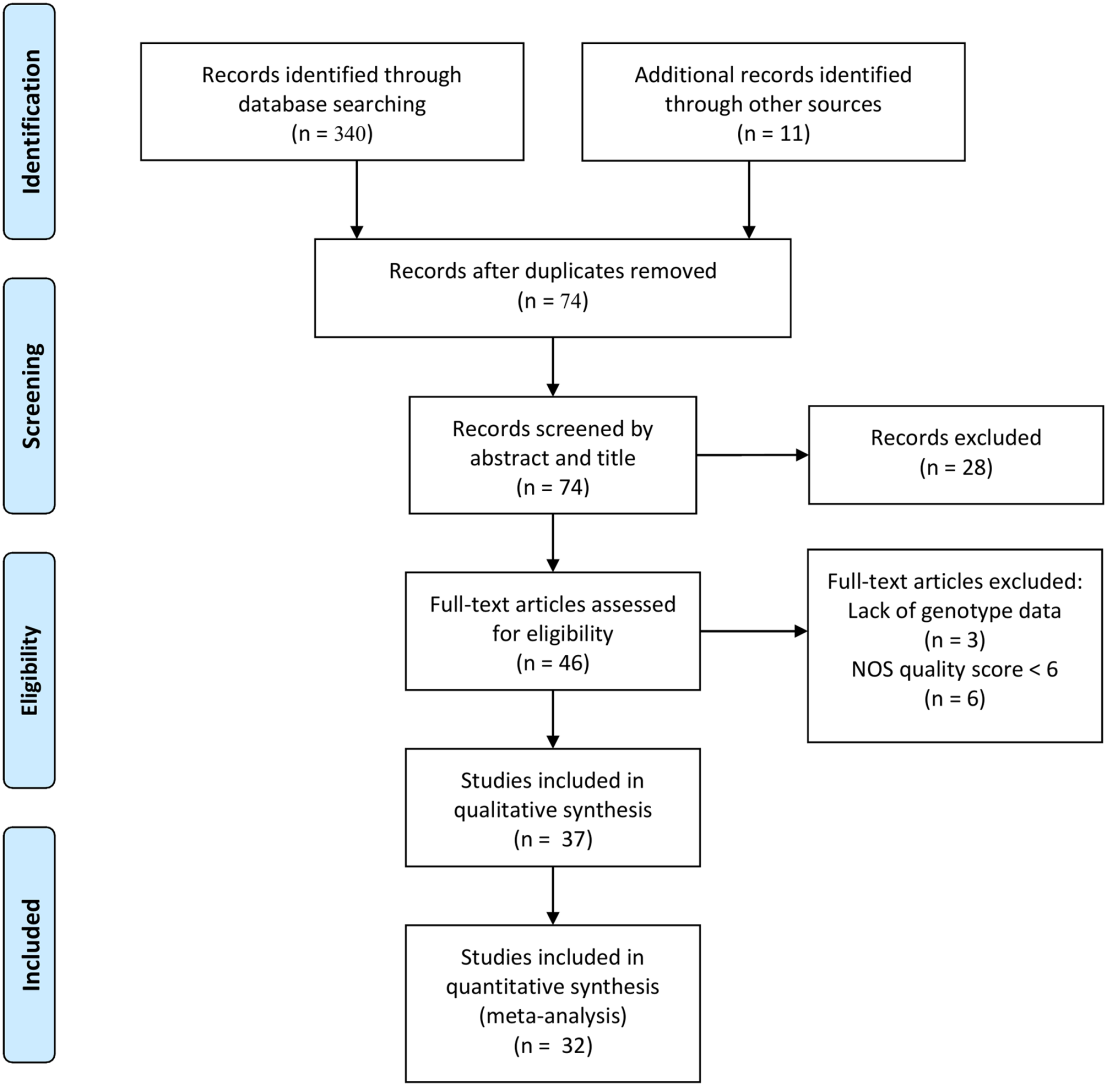
Flowchart showing the study selection procedure for identified and included articles.

The search using PubMed, Medline and EMBASE databases yielded 351 articles (Fig 1). Of these, 277 were duplicates and removed, with the remaining 74 articles screened by title and abstract. Articles (n = 28) were excluded that were not in English, did not deal with TLRs or TB disease or were previous meta-analyses or review articles. The remaining 46 articles were thoroughly reviewed, and 3 were excluded due to lack of genotype data, and a further 6 were excluded due to low NOS quality scores (S1 Table). Data was extracted from the remaining 37 studies. A further 5 studies were excluded due to the genotype data in the control group not being in HWE (*p*-value < 0.05), resulting in 32 articles (Table 1) being analysed in this meta-analysis. Excluded articles are listed in S2 Table. Genotype and allele counts for all included studies are listed in S3 Table, as well as the calculated HWE *p*-values. Genome wide association studies (GWAS) were also considered, but no GWAS study found an association between *TLR* polymorphisms and TB susceptibility and data was not available for the SNPs analysed in this meta-analysis.

**Table 1:** Characteristics of the 32 studies included in this meta-analysis, grouped by TLR gene, SNP and ethnicity.

Gene	SNP	Author and year	Population	Ethnicity	Cases	Controls	NOS score
***TLR1***	rs4833095	Sinha et al., 2014[20]	North Indian	Asian	204	126	8
		Qi et al., 2015[21]	Chinese	Asian	340	366	7
		Dittrich et al., 2015[22]	India	Asian	206	239	6
		Salie et al., 2015[23]	SAC	African	324	344	8
		Ma et al., 2007[24]	African American	African	339	194	7
		Ma et al., 2007[24]	American Caucasian	European	180	110	7
		Ma et al., 2007[24]	Hispanic	Hispanic	375	114	7
	rs5743618	Sinha et al., 2014[20]	North Indian	Asian	160	124	8
		Ma et al., 2010[25]	Chinese	Asian	543	544	8
		Selvaraj et al., 2010[26]	South India	Asian	206	212	7
		Qi et al., 2015[21]	Chinese	Asian	340	366	7
		Wu et al., 2015[27]	Chinese	Asian	109	422	8
		Salie et al., 2015[23]	SAC	African	328	330	8
		Ma et al., 2007[24]	African American	African	339	194	7
		Ocejo-Vinyals et al., 2013[28]	Spanish Caucasian	European	190	192	8
		Ma et al., 2007[24]	American Caucasian	European	180	110	7
		Ma et al., 2007[24]	Hispanic	Hispanic	375	114	7
***TLR2***	rs3804099	Caws et al., 2008[29]	Vietnam	Asian	187	237	6
		Yang et al., 2013[30]	China	Asian	200	196	8
		Wu et al., 2015[27]	Chinese	Asian	109	422	8
		Salie et al., 2015[23]	SAC	African	435	292	8
		Arji et al., 2014[31]	Morocco	African	343	202	7
		Ma et al., 2007[24]	African American	African	339	194	7
		Etokebe et al., 2010[32]	Croatia Caucasian	European	190	489	6
		Ma et al., 2007[24]	American Caucasian	European	180	110	7
		Torres-García et al., 2013[33]	Mexican	Hispanic	90	90	8
		Sánchez et al., 2012[8]	Columbia	Hispanic	465	300	8
		Ma et al., 2007[24]	Hispanic	Hispanic	375	114	7
	rs5743708	Selvaraj et al., 2010[26]	South India	Asian	206	212	7
		Xue et al., 2010[34]	Chinese	Asian	205	203	7
		Wu et al., 2015[27]	Chinese	Asian	103	418	8
		Salie et al., 2015[23]	SAC	African	438	288	8
		Ma et al., 2007[24]	African American	African	339	194	7
		Dalgic et al.(b), 2011[35]	Turkish	European	138	200	7
		Etokebe et al., 2010[32]	Croatia Caucasian	European	103	105	6
		Ma et al., 2007[24]	American Caucasian	European	180	110	7
		Torres-García et al., 2013[33]	Mexican	Hispanic	90	90	8
		Sánchez et al., 2012[8]	Columbia	Hispanic	466	300	8
		Ma et al., 2007[24]	Hispanic	Hispanic	375	114	7
	rs3804100	Wu et al., 2015[27]	Chinese	Asian	109	422	8
		Chen et al., 2010[36]	Taiwan	Asian	184	184	7
		Salie et al., 2015[23]	SAC	African	435	292	8
		Ma et al., 2007[24]	African American	African	339	194	7
		Ma et al., 2007[24]	American Caucasian	European	180	110	7
		Etokebe et al., 2010[32]	Croatia Caucasian	European	186	551	6
		Ma et al., 2007[24]	Hispanic	Hispanic	375	114	7
	GT(n)	Xue et al., 2010[34]	Chinese	Asian	244	233	7
		Chen et al., 2010[36]	Taiwan	Asian	367	368	7
		Yim et al., 2006[37]	Korean	Asian	516	382	6
		Salie et al., 2015[23]	SAC	African	345	242	8
***TLR4***	rs4986790	Jahantigh et al., 2013[3]	South East Iran	Asian	124	149	7
		Selvaraj et al., 2010[26]	South India	Asian	206	212	7
		Najmi et al., 2010[11]	Indian	Asian	135	250	7
		Wu et al., 2015[27]	Chinese	Asian	109	422	8
		Salie et al., 2015[23]	SAC	African	421	287	8
		Ma et al., 2007[24]	African American	African	339	194	7
		Fitness et al., 2004[38]	Malawi	African	162	427	7
		Olesen et al., 2007[39]	West African	African	315	337	7
		Ma et al., 2007[24]	American Caucasian	European	180	110	7
		Rosas-Taraco et al., 2007[40]	Mexican	Hispanic	104	114	7
		Torres-García et al., 2013[33]	Mexican	Hispanic	90	90	8
		Ma et al., 2007[24]	Hispanic	Hispanic	375	114	7
		Sánchez et al., 2012[8]	Columbian	Hispanic	466	300	8
	rs4986791	Najmi et al., 2010[11]	Indian	Asian	135	205	7
		Jahantigh et al., 2013[3]	South East Iran	Asian	124	149	7
		Selvaraj et al., 2010[26]	South India	Asian	203	203	7
		Wu et al., 2015[27]	Chinese	Asian	109	422	8
		Salie et al., 2015[23]	SAC	African	439	292	8
		Ma et al., 2007[24]	African American	African	339	194	7
		Ma et al., 2007[24]	American Caucasian	European	180	110	7
		Ma et al., 2007[24]	Hispanic	Hispanic	375	114	7
		Sánchez et al., 2012[8]	Columbian	Hispanic	466	300	8
***TLR 6***	rs5743810	Sinha et al., 2014[20]	North Indian	Asian	204	124	8
		Selvaraj et al., 2010[26]	South India	Asian	206	212	7
		Wu et al., 2015[27]	Chinese	Asian	109	422	8
		Ma et al., 2007[24]	African American	African	339	194	7
		Ma et al., 2007[24]	American Caucasian	European	180	110	7
		Ma et al., 2007[24]	Hispanic	Hispanic	375	114	7
***TLR8*** *	rs3764879	Davila et al., 2008[41]	Indonesian	Asian	222	225	6
	Males	Salie et al., 2015[23]	SAC	African	408	194	8
		Dalgic et al., 2011[42]	Turkish	European	62	72	7
		Davila et al., 2008[41]	Russian	European	1341	1308	6
	rs3764879	Davila et al., 2008[41]	Indonesian	Asian	280	304	6
	Females	Salie et al., 2015[23]	SAC	African	220	334	8
		Dalgic et al., 2011[42]	Turkish	European	62	78	7
	rs3764880	Hashemi-Shahri et al., 2014[43]	Iran	Asian	77	62	7
	Males	Davila et al., 2008[41]	Indonesian	Asian	222	225	6
		Bukhari et al., 2015[44]	Pakistan	Asian	45	22	7
		Salie et al., 2015[23]	SAC	African	372	162	8
		Davila et al., 2008[41]	Russian	European	1341	1308	6
		Dalgic et al., 2011[42]	Turkish	European	62	72	7
	rs3764880	Hashemi-Shahri et al., 2014[43]	Iran	Asian	98	83	7
	Females	Davila et al., 2008[41]	Indonesian	Asian	280	304	6
		Bukhari et al., 2015[44]	Pakistan	Asian	58	65	7
		Salie et al., 2015[23]	SAC	African	199	306	8
		Davila et al., 2008[41]	Russian	European	1341	1308	6
		Dalgic et al., 2011[42]	Turkish	European	62	78	7
***TLR9***	rs352139	Kobayashi et al., 2012[45]	Indonesia	Asian	537	560	8
		Kobayashi et al., 2012[45]	Vietnam	Asian	276	455	8
		Salie et al., 2015[23]	SAC	African	427	440	8
		Torres-García et al., 2013[33]	Mexican	Hispanic	90	90	8
	rs5743836	Selvaraj et al., 2010[26]	South India	Asian	206	212	7
		Olesen et al., 2007[39]	West African	African	321	347	7
		Wu et al., 2015[27]	Chinese	Asian	109	422	8
		Salie et al., 2015[23]	SAC	African	431	435	8
		Torres-García et al., 2013[33]	Mexican	Hispanic	90	90	8
	rs1870884	Selvaraj et al., 2010[26]	South India	Asian	193	208	7
		Jahantigh et al., 2013[3]	South East Iran	Asian	124	149	7
		Wu et al., 2015[27]	Chinese	Asian	109	422	8
		Olesen et al., 2007[39]	West African	African	318	339	7

*TLR8 is on the X-chromosome and all analysis for these SNPs were done separately in males and females.

### Meta-analysis results

We analysed 14 SNPs in this meta-analysis, of which eight (*TLR1* rs5743618 [20,21,23–28], *TLR2* rs3804099 [8,23,24,27,29–33] and rs5743708 [8,23,24,26,27,32–35], *TLR4* rs4986790 [3,8,11,23,24,26,27,33,38–40] and rs4986791 [3,8,11,23,24,26,27], *TLR6* rs5743810 [20,24,26,27], *TLR9* rs1870884 [3,26,27,39] and GT(n) repeats [23,34,36,37]) had sufficient studies available to perform subgroup analysis on at least one ethnic group (African, European, Asian and/or Hispanic). The *TLR8* SNPs rs3764879 [23,41,42] and rs3764880 [23,41–44] are located on the X chromosome and were analysed in a sex-stratified manner, with no associations identified across the populations with regards to TB susceptibility. A significant association with TB susceptibility was found for 6 *TLR* SNPs. A summary of the meta-analysis as well as Egger’s test for publication bias results can be found in the S4 Table. Forest plots for non-significant associations can be found in S1 Fig.

#### *TLR1* rs4833095

**Fig 2:**
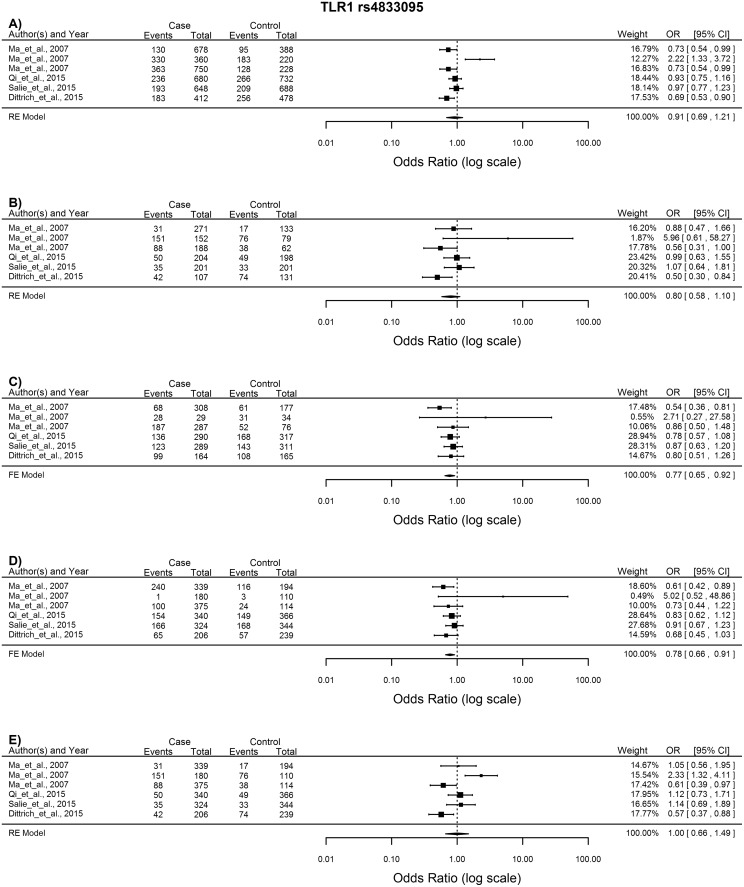
Forest plot of the association between *TLR1* rs4833095 and TB risk for all five models. A) Allelic model, B) Homozygote comparison, C) Heterozygote comparison, D) Dominant model, E) Recessive model. OR: odds ratio; 95%CI: 95% confidence interval.

Six studies were included in the analysis of the *TLR1* rs4833095 SNP using the FE model given that minimal heterogeneity existed between the studies. The heterozygous comparison showed decreased susceptibility to TB for individuals with the AG genotype (AG vs. GG: OR = 0.77, 95% CI = 0.65–0.95, p = 0.0031). The dominant model also showed a decreased susceptibility to TB with the AA plus AG genotype (AA +AG vs. GG: OR = 0.78, 95% CI = 0.66–0.91, p = 0.0021) indicating that the AG genotype might exert a protective effect (Fig 2).

#### *TLR2* rs5743708

**Fig 3:**
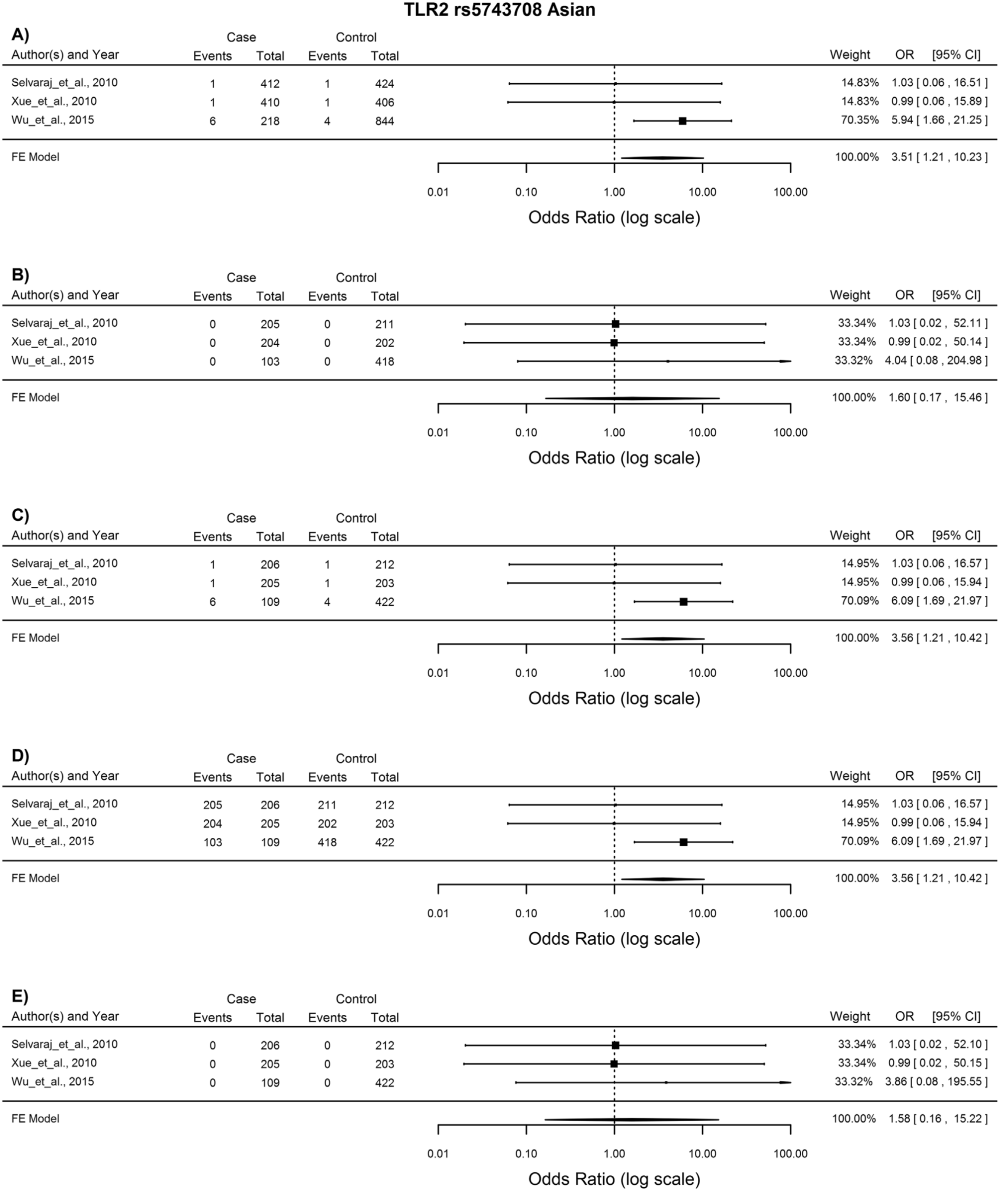
Forest plot of the association between *TLR2* rs5743708 and TB risk for all five models in the Asian subgroup. A) Allelic model, B) Homozygote comparison, C) Heterozygote comparison, D) Dominant model, E) Recessive model. OR: odds ratio; 95%CI: 95% confidence interval.

**Fig 4:**
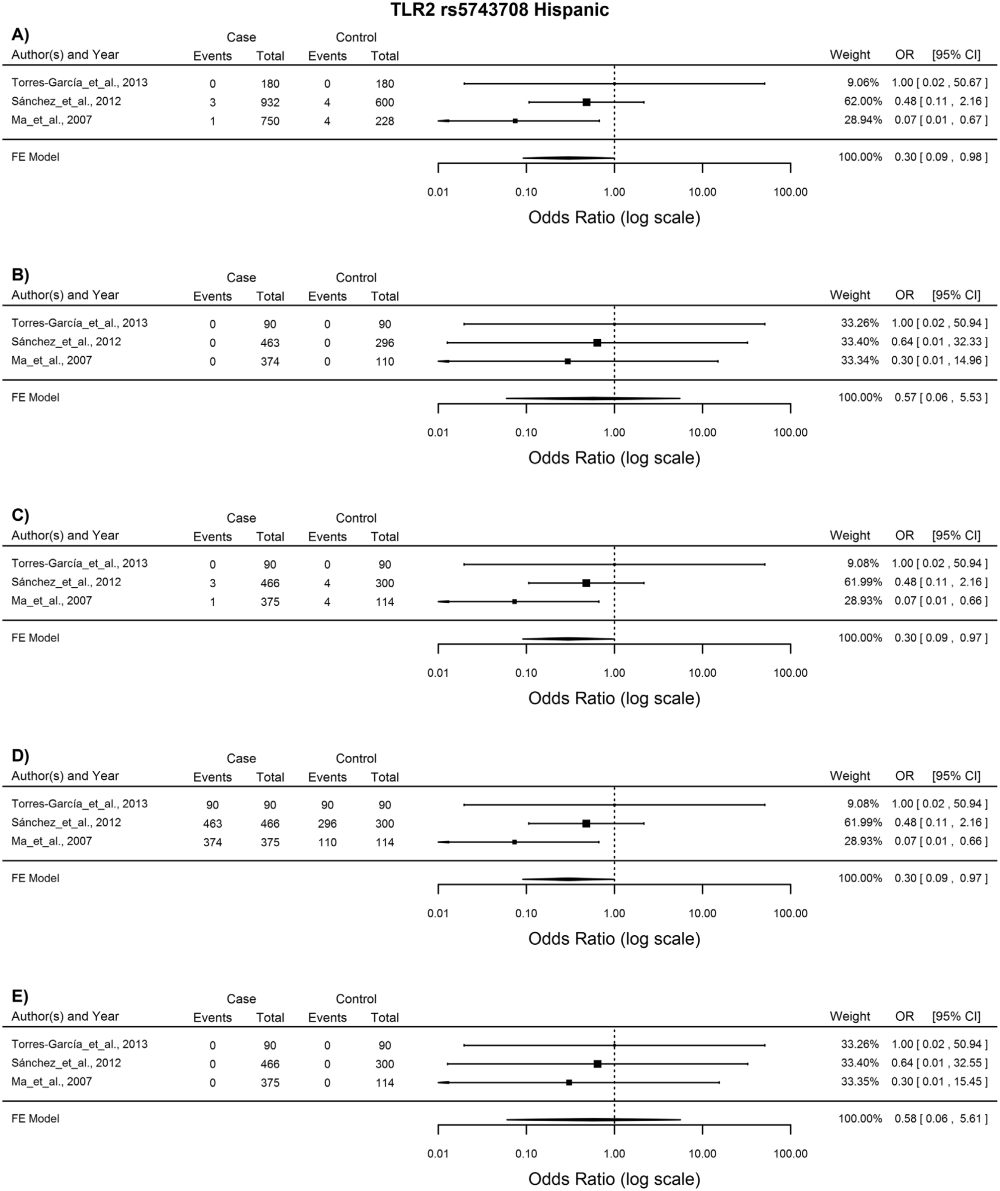
Forest plot of the association between *TLR2* rs5743708 and TB risk for all five models in the Hispanic subgroup. A) Allelic model, B) Homozygote comparison, C) Heterozygote comparison, D) Dominant model, E) Recessive model. OR: odds ratio; 95%CI: 95% confidence interval.

The meta-analysis of the 11 studies included for the analysis of this SNP showed no association with TB susceptibility with any of the analytical models. However subgroup analysis showed association with TB susceptibility with the allelic, heterozygote and dominant models in the Asian ethnic group (A vs. G: OR = 3.51, 96%CI = 1.21–10.32, p = 0.021; AG vs. GG: OR = 3.56, 95%CI = 1.21–10.42, p = 0.021; AA vs. AG + GG: OR = 3.56, 95%CI = 1.21–10.42, p = 0.021) and Hispanic group (A vs. G: OR = 0.3, 96%CI = 0.09–0.98, p = 0.046; AG vs. GG: OR = 0.3, 95%CI = 0.09–0.97, p = 0.045; AA vs. AG + GG: OR = 0.3, 95%CI = 0.09–0.97, p = 0.045). Three studies were included for each ethnic group and statistical analysis was done using the FE model. In the Asian population the A allele increased susceptibility to TB (Fig 3), while in the Hispanic population it conferred protection against TB disease (Fig 4).

#### *TLR2* rs3804100

**Fig 5:**
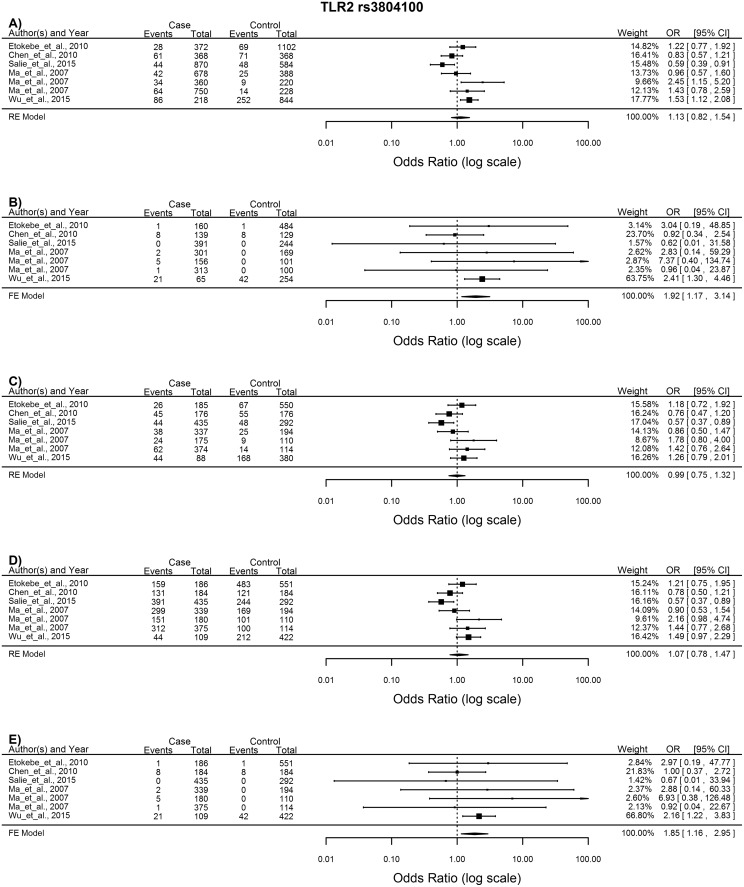
Forest plot of the association between *TLR2* rs3804100 and TB risk for all five models. A) Allelic model, B) Homozygote comparison, C) Heterozygote comparison, D) Dominant model, E) Recessive model. OR: odds ratio; 95%CI: 95% confidence interval.

Little heterogeneity existed between the 7 studies included in the meta-analysis of this SNP and thus the FE model was used to analyse the homozygote and recessive models, both of which indicated that the CC genotype increases the risk of developing TB (CC vs. TT: OR = 1.92, 95%CI = 1.17–3.14, p = 0.009; CC vs. TC + TT: OR = 1.85, 95%CI = 01.16–2.95, p = 0.01) (Fig 5).

#### *TLR4* rs4986791

**Fig 6:**
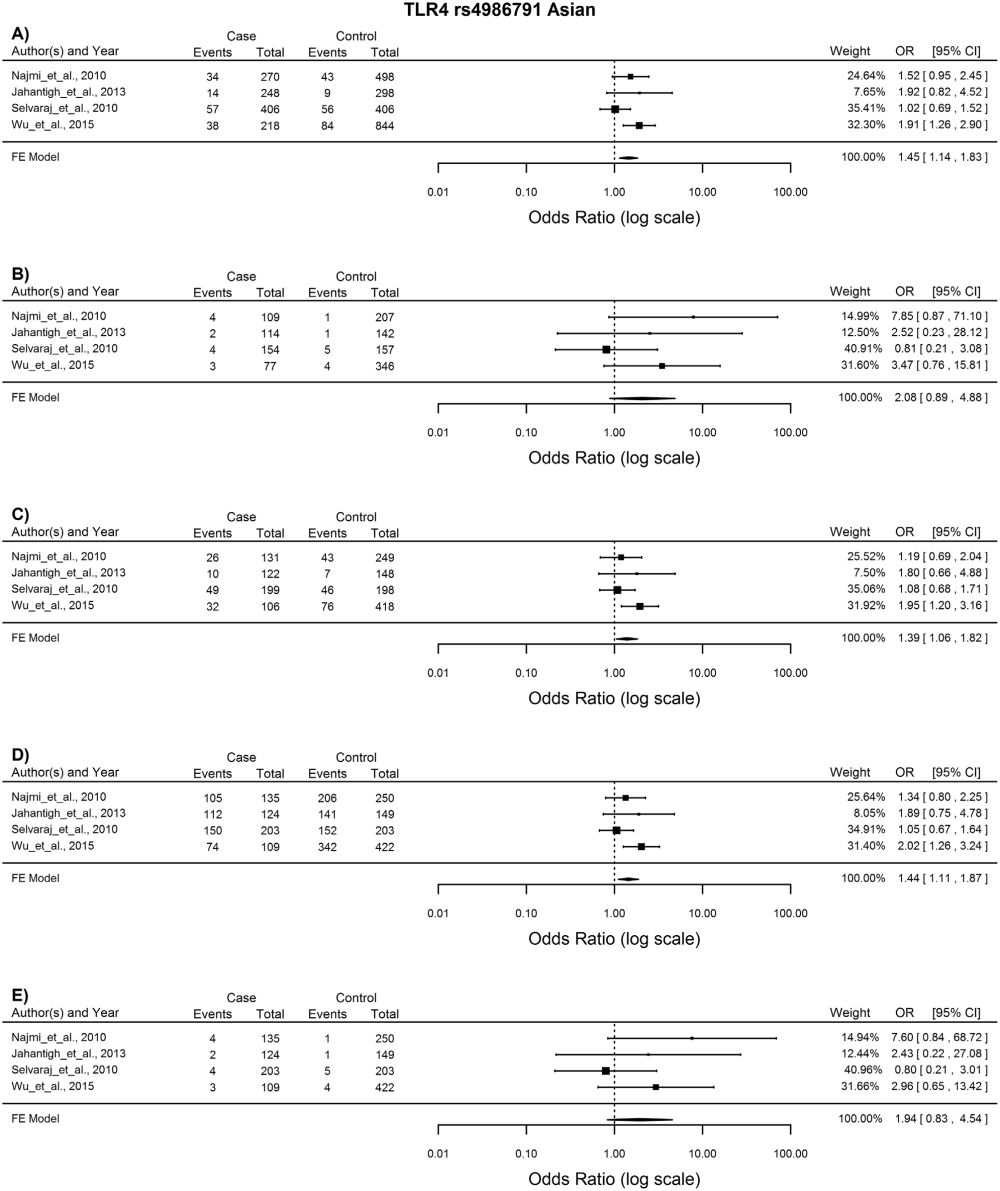
Forest plot of the association between *TLR4* rs4986791 and TB risk for all five models in the Asian subgroup. A) Allelic model, B) Homozygote comparison, C) Heterozygote comparison, D) Dominant model, E) Recessive model. OR: odds ratio; 95%CI: 95% confidence interval.

Nine studies were included in the meta-analysis of this SNP, but no significant association with TB susceptibility was found overall. In the subgroup analysis of the Asian population, which included 4 studies, the T allele, TC and TT genotypes were associated with increased susceptibility to TB in the allelic, heterozygous and dominant models, all of which were analysed using the FE model as very little heterogeneity was observed (T vs. C: OR = 1.45, 95%CI = 1.14–1.83, p = 0.002; TC vs. CC: OR = 1.39, 95%CI = 1.06–1.82, p = 0.019; TT + TC vs. CC: OR = 1.44, 95%CI = 1.11–1.87, p = 0.007) (Fig 6).

#### *TLR6* rs5743810

**Fig 7:**
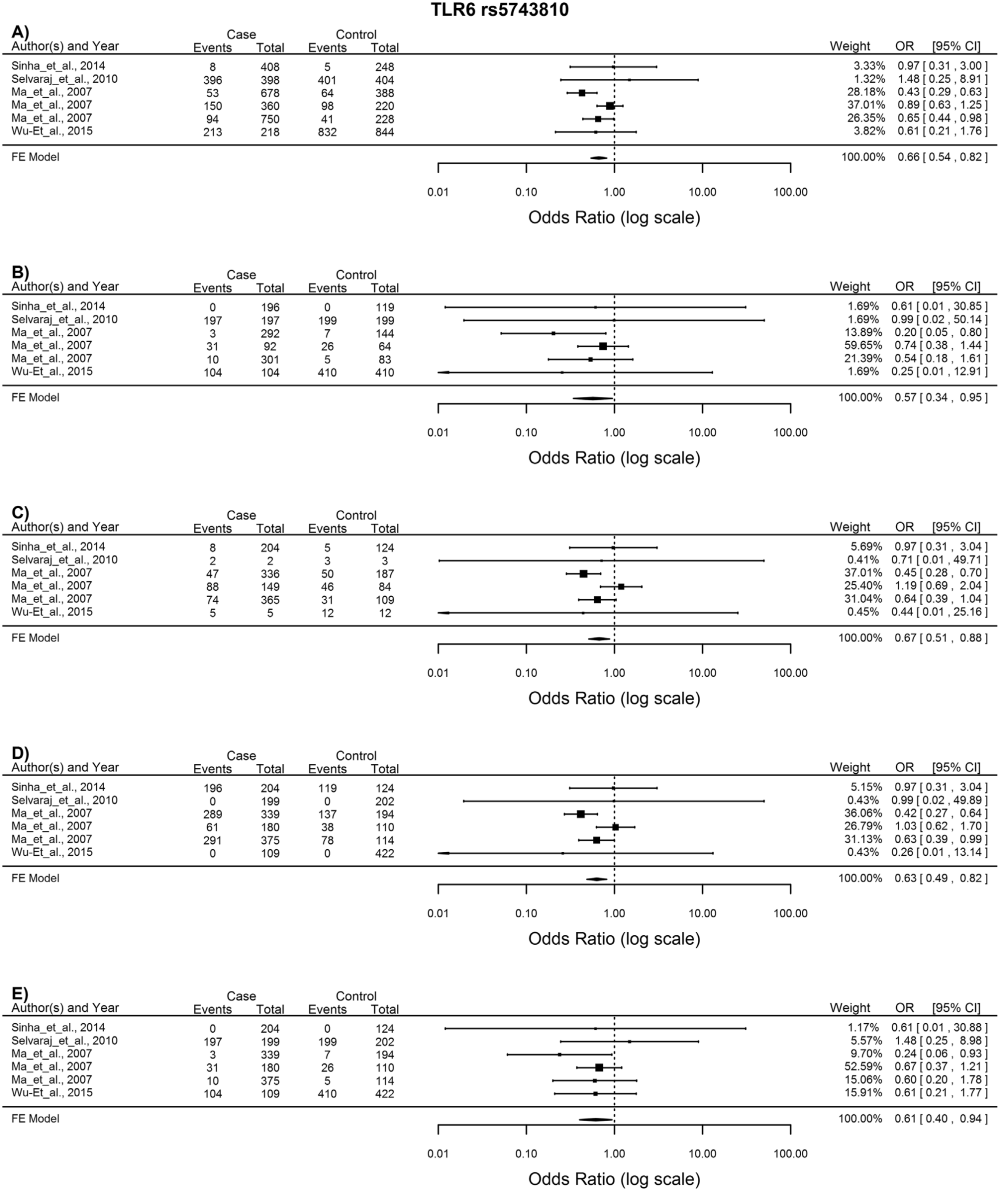
Forest plot of the association between *TLR6* rs5743810 and TB risk for all five models. A) Allelic model, B) Homozygote comparison, C) Heterozygote comparison, D) Dominant model, E) Recessive model. OR: odds ratio; 95%CI: 95% confidence interval.

The 4 articles included for the meta-analysis of this SNP showed no significant heterogeneity. The T allele conferred protection against TB in the allelic model (T vs. C: OR = 0.66, 95%CI = 0.54–0.82, p = 0.0001) and the TT and TC genotypes also had a protective effect against TB infection in the homozygote, heterozygote, dominant and recessive models (TT vs. CC: OR = 0.57, 95%CI = 0.34–0.95, p = 0.03; TC vs. CC: OR = 0.67, 95%CI = 0.51–0.88, p = 0.004; TT + TC vs. CC: OR = 0.63, 95%CI = 0.49–0.82, p = 0.0005; TT vs. TC + CC: OR = 0.61, 95%CI = 0.4–0.94, p = 0.024) (Fig 7). Subgroup analysis on the Asian ethnic group showed no significant results.

#### *TLR9* rs352139

**Fig 8:**
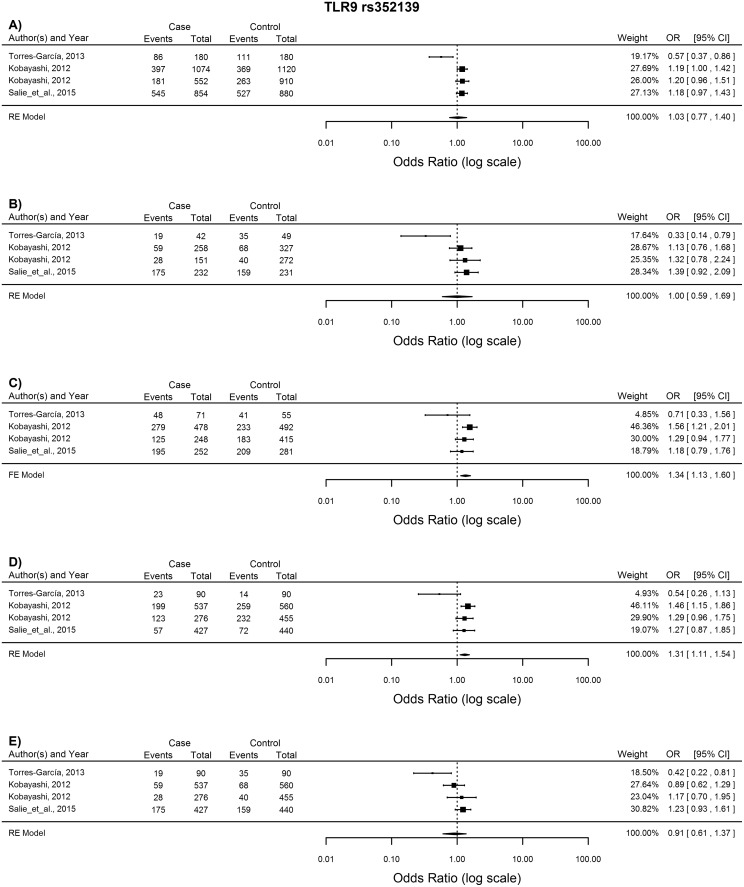
Forest plot of the association between *TLR9* rs352139 and TB risk for all five models. A) Allelic model, B) Homozygote comparison, C) Heterozygote comparison, D) Dominant model, E) Recessive model. OR: odds ratio; 95%CI: 95% confidence interval.

Four studies were included in the analysis of this SNP. The GA and GG genotypes for the heterozygous and dominant comparison were associated with increased susceptibility to TB when analysed using the FE and RE models, respectively (GA vs. AA: OR = 1.34. 95% CI = 1.13–1.60, p = 0.0008; GG vs. GA + AA: OR = 1.31, 95%CI = 1.11–1.54, p = 0.0015) (Fig 8).

### Publication bias and sensitivity analysis

The results of the Egger’s test (S4 Table) showed evidence of publication bias for only one SNP. Both the meta-analysis on *TLR1* rs5743618 and its subgroup analysis on the Asian population showed publication bias for the allelic, heterozygote and dominant model. However, correcting for this bias did not change the significance of the results (S4 Table).

For the sensitivity analysis, studies were sequentially excluded from the meta-analysis in order to assess the influence of each study on the pooled results (OR, 95% CI and p-value) of each model for each SNP. However, the pooled results did not significantly differ for any SNP regardless of the statistical model used.

## Discussion

The influence of TLRs on TB susceptibility has been extensively investigated and while often associated, replication in different ethnic groups has yielded contradictory results [24]. One factor contributing to this variability is the power of the individual studies which is limited by sample size. This meta-analysis was performed to aggregate information across studies using statistical measures for the most commonly investigated *TLR* SNPs in order to increase the sample size and in turn the power to find or disprove previous associations. Furthermore, meta-analysis gives an indication of which *TLR* SNPs are associated with TB susceptibility across ethnicities and which are specific to a certain ethnic group. For this meta-analysis 14 of the most commonly investigated *TLR* SNPs were analysed of which five were associated with TB disease susceptibility across different ethnic groups. Seven of the 14 analysed SNPs had enough data available for subgroup analysis of which two showed associations in subgroup analysis but not across different ethnic groups.

The AG genotype of *TLR1* rs4833095 was associated with resistance to TB across ethnic groups in this meta-analysis. This non-synonymous polymorphism, located in the extracellular domain, results in an asparagine to serine amino acid change [24]. This amino acid change is thought to affect the folding mechanism of the TLR and its ligand binding efficiency as well as impair its ability to form heterodimers with TLR2, thus leading to a reduced immune response [24]. *TLR1* rs4833095 is in strong linkage disequilibrium (LD) with *TLR1* rs5743618 and determining how these two SNPs contribute to TB susceptibility has proven difficult. Studies in HEK293 cells have shown that the GG genotype of rs5743618 resulted in decreased NF-kB expression, while the presence of any of the rs4833095 alleles did not further affect NF-kB levels [46]. Uciechowski *et al*. [5] proposed that this polymorphism affects cell surface expression of TLR1 as it is involved in the transport of the PRR to the cell surface, while rs5743618 is thought to affect signal transduction of TLR1. The fact that these two SNPs are in LD means that expression and signalling of TLR1 is affected, which can have a major impact on the immune system and requires gene-gene interaction studies to fully validate. Furthermore, rs5743618 did not show any association with TB susceptibility, neither across populations nor in the Asian ethnic subgroup.

The AG [20] and AA [22] genotypes of rs4833095 have been proposed to confer protection against TB, which agrees with this meta-analysis. No previous meta-analyses have been conducted on *TLR1* rs4833095, but Zhang *et al*. [7] conducted a meta-analysis on *TLR1* rs5743618 and also found no association with TB susceptibility, which corresponds with our results. Our study had greater power to detect associations due to an increased sample size (2769 cases and 2625 controls vs. 1648 cases and 1216 controls).

In the meta-analysis of *TLR2* rs5743708 across populations no association with TB susceptibility was found, but in the subgroup analysis of the Asian and Hispanic populations the AA and AG genotypes were significantly associated with TB disease susceptibility, as found previously [47, 27]. This non-synonymous arginine to glutamine substitution in the Toll-Interleukin–1 receptor (TIR) domain of the receptor has been proposed to result in a decreased response of macrophages to bacterial peptides [47], and studies in mice have shown that rs5743708 alters TLR2 signalling leading to lower levels of tumour necrosis factor (TNF)α and interferon-gamma (IFN-γ) and a diminished immune response [48]. TNFα and IFN-γ work in synergy to activate macrophages, which leads to the production of reactive nitrogen intermediates (RNI) [49]. These RNI’s mediate the macrophages’ ability to control *M*. *tuberculosis* proliferation as well as stimulating the migration of immune cells to the site of infection. Furthermore, TLR2 has been shown to form heterodimers with TLR1 and TLR6 to recognise a wide variety of ligands [48] and thus polymorphisms leading to defective TLR2 activation can affect multiple PRRs and have a compounded negative effect on the immune system.

Three meta-analyses have previously been done on *TLR2* rs5743708 yielding similar results. Sun *et al*. [50] found that the AA genotype was associated with increased TB risk across populations in the allelic, heterozygous and dominant model. Similarly, Wang *et al*. [51] found that the AA genotype and A allele, in the dominant and allelic model, increased TB susceptibility across different ethnic groups and was significantly associated in the Asian, but not European subgroup. Finally, Zhang *et al*. [7] showed a significant association with TB susceptibility for the A allele and AA genotype across different ethnic groups, and subgroup analysis for the allelic model showed increased risk in Asians and Europeans, but decreased risk in the Hispanic population. The results from these previous meta-analyses agree to some extent with our results as they also show increased susceptibility in the Asian population, but a protective effect in the Hispanic population, and did not show association with TB susceptibility across ethnic groups. The reason for this lack of global association could be the fact that the previous meta-analysis had more Asian cohort studies which led to across population associations. The very low or sometimes absent minor allele frequencies of this SNP can also influence the results as the frequencies differ between the ethnic groups.

*TLR2* rs3804100, which represents a synonymous serine to serine amino acid substitution at position 450 in the extracellular domain of TLR2, has to date not been fully investigated and the functional effects of this SNP are still uncertain. The CC genotype of this polymorphism is associated with latent TB infection but not with active pTB disease [27], is associated with higher blood natural killer cell counts and is thought to predispose pTB patients to the development of systemic symptoms or pleural involvement [36]. This coincides with our results which showed that the CC genotype increases susceptibility to TB. Two previous meta-analysis have been performed on this polymorphism and no association with TB susceptibility was found in either analysis. Zhang *et al*. [7] had a smaller sample size (1379 cases and 1309 controls vs. 1808 cases and 1867 controls) which could explain the contradictory results. The second meta-analysis by Sun *et al*. [50] however had very similar sample sizes to ours (1873 cases and 1954 controls vs. 1808 cases and 1867 controls) and it is thus unlikely that sample size is a cause for the lack of association. However, the meta-analysis by Sun *et al*. [50] included two studies that are not included in our meta-analysis and given that both of these studies come from an Asian cohort, a protective effect of the CC genotype in the Asian population could obscure the results.

There was no global association with TB susceptibility observed for *TLR4* rs4986791, but subgroup analysis in the Asian population showed increased susceptibility to TB. This missense variant results in an amino acid substitution of threonine with isoleucine in the extracellular domain of TLR4 and is thought to alter a co-receptor binding region affecting the ability of the receptor to induce signalling in response to LPS stimulation in some cell types [3]. Previously, the TT and TC genotypes of this SNP have been associated with increased TB risk in Asian populations [11,27], which corroborates the results of our study. Two smaller meta-analysis on this SNP also found no cross population association with TB, but subgroup analysis could not be performed as a limited number of studies were available [50,52].

The overall trend for the *TLR6* rs5743810 SNP is that the T allele, and the TC and TT genotypes have a protective effect against TB development. This non-synonymous polymorphism results in an amino acid change from proline to serine in the extracellular domain and has been proposed to influence ligand recognition and reduced signal response [53]. The T allele was found by Shey *et al*. [53] to reduce NF-kB signalling which led to an altered level of IL–6 production, while Randhawa *et al*. [54] showed that it leads to increased IFN-γ production and thus protection against *M*. *tuberculosis*. These functional studies correlate with the results found in this meta-analysis as well as that of Zhang *et al*. [7] where the T allele and TT genotype was also associated with resistance to TB disease.

The intracellular TLR9 molecule recognises unmethylated CpG motifs in mycobacterial DNA [45] and in TLR9 deficient mice, was vital for activation of the Th1 immune response [55]. Tao *et al*. [56] proposed that this intronic polymorphism (rs352139) either directly affects the expression of the *TLR9* gene, or is in LD with a polymorphic regulatory region that controls *TLR9* expression. A second hypothesis is that this intronic variant could introduce alternative splice sites, affecting the TLR9 mRNA transcript and thus the structure and signalling capability of this PRR, leading to an altered immune response [41]. The results indicate that the GG and GA genotype of this *TLR9* rs352139 polymorphism might influence TB susceptibility, coinciding with previous results [56, 57, 58].

The functional effects for most of the associated *TLR* SNPs identified in this meta-analysis have not been fully investigated. Given that the majority of these SNPs result in structural changes (non-synonymous or splice-site variants) it could cause altered PRR signalling and efficacy. Impaired signal transduction could lead to reduction of activated transcription factors (e.g. NF-kB) and thus altered levels of pro and anti-inflammatory cytokines, nitric oxide, chemokines and interferon (IFN) inducible genes [59]. An effective immune response requires a balance between these chemokines and cytokines and any disruption of this equilibrium could have a detrimental effect on the immune system and thus increase susceptibility to TB [60].

While general trends were observed in about a third of the SNPs analysed, the effects of most *TLR* polymorphisms on disease susceptibility seem to be population dependent. This lack of association across ethnic groups may be due to the large variability in allelic distribution between ethnic groups, which may in part be explained by evolutionary pressures. In Europe, the epidemic proportions of TB during the industrial revolution could have led to natural selection and thus the accumulation of SNPs that protected against TB. Conversely, the African populations encountered high levels of TB only at a much later stage and would have had less time to accumulate protective polymorphisms [61]. SNPs associated with TB susceptibility in specific ethnicities had MAFs that differed between the ethnic groups. In the case of *TLR2* rs5743708 the minor allele was present in the Asian ethnic group only in cases (MAF = 0.03), while in the Hispanic ethnic group the minor allele was present only in controls (MAF = 0.015). This could have led to the opposite effect detected for TB susceptibility in these two ethnic groups. In the 1000 genomes project [62] the variant is monomorphic in all Asian populations and occurs only at very low frequencies (MAF 0.01) in two of the four Hispanic subgroups. The dbSNP database indicated that the minor allele is present in the Hispanics (MAF = 0.087) and central Asian population (MAF = 0.062), but absent in the East Asian population [63]. This corresponds with our data as we included studies from both East and central Asia. It is also possible that the opposing effect is due to a causative variant in LD with *TLR2* rs5743708, as was observed for monocyte chemoattractant protein–1 (*MCP–1)* gene polymorphisms [64]. For *TLR4* rs4986791 the Asian population was the only ethnic group that showed a significant difference between cases (MAF = 0.125) and controls (MAF = 0.09) (S3 Table). However, the 1000 genomes project [62] shows that the T allele was completely absent or present only at low frequencies (MAF < 0.07) in most ethnic groups except for the South Asians(MAF = 0.12) suggesting a population-specific association. This corresponds with our data as three of the four Asian population studies included for this SNP were South Asian (Table 1). Furthermore, host-strain interactions add to the complexity of the disease as the phenotypic and genetic characteristics of the infecting mycobacterial strain can have varying effects on disease outcome depending on the genotypic makeup of the host [65].

The lack of associations across ethnic groups could be due to the limitations of this meta-analysis. Firstly, the number of studies available that investigate the association between TLRs and TB susceptibility is limited. The majority of SNPs investigated have only one or two studies and as one of the inclusion criteria was that at least three studies be available for the meta-analysis, a large number of variants could not be investigated. The lack of studies also meant that for many of the SNPs investigated even the pooled sample size was limited, lowering the power to find an association, especially for subgroup analysis. Furthermore, there are many more types of PRRs (NLR, RLR, and CLR) that are involved in the recognition of *M*. *tuberculosis* and protection against TB that were not investigated in this meta-analysis, which could also have gene-gene interaction effects that were not possible to investigate here. Finally data on confounding factors such as age, gender and smoking, which may differ between cases and controls and between studies, was mostly not available and thus could not be corrected for using meta-regression analysis. These confounding effects could generate false findings (positive confounding) or obscure true associations (negative confounding) and could thus influence the results [66].

Obtaining across or within ethnicity-specific information about the effect on TB susceptibility due to genetic variations in the innate immune system could have valuable applications in “host-directed therapies” [67] or translational research. As the innate immune response fails to control *M*. *tuberculosis* infection if it is either excessive or inadequate [68], a deeper understanding of the effect that various polymorphisms have on TB susceptibility is vital for the development of host-directed therapies. The results of meta-analyses such as this could help set up treatments, global- and population-specific, to maintain the innate immune response between the two extremes and increase resistance to *M*. *tuberculosis* infection.

Given the lack of association between TLR and TB, it is clear that although TLRs may be critical for the defence against *M*. *tuberculosis*, other PRRs and gene-gene interactions should also be investigated. There is a measure of redundancy in the immune system as one type of PAMP can trigger multiple PRRs and lead to the activation of similar immune pathways. Additional studies with larger sample sizes and ethnic variety should be conducted on all PRRs involved in *M*. *tuberculosis* detection to shed some light on this complex disease and for the data to be usable in the medical field.

Results from GWAS investigating TB susceptibility do not coincide with results from this and other meta-analysis [69–72]. This could be due to the complexity and multifactorial nature of TB disease. The genetic aetiology of TB susceptibility may be explained by several genetic variants having a small effect on disease outcome. These variants would therefore not reach significance in GWAS due to the burden of correcting for multiple testing. Furthermore, little to no consistency has been noted across the TB GWAS’s published to date.

In summary, this meta-analysis aimed to summarize the effects of the most commonly investigated *TLR* SNPs in relation to TB susceptibility. We found that the majority of SNPs showed no association in general or in ethnic subgroup analysis. Only four SNPs (rs4833095, rs3804100, rs5743810 and rs352139) showed significant general associations and two others showed significant subgroup associations (rs5743708 and rs4986791). While this meta-analysis gives an overview of the effect of *TLR* SNPs on TB disease susceptibility, more studies in various ethnic groups need to be done in order to reinforce the results of this meta-analysis and fully elucidate which variants are population-specific and which have a general association with TB susceptibility.

## Supporting Information

S1 Fig Forest plots for all non-significantly associated SNPs including subgroup analysis. A) Allelic model, Ai) Allelic model following D&T correction, B) Homozygote comparison, C) Heterozygote comparison, Ci) Heterozygote comparison following D&T correction, D) Dominant model, Di) Dominant model following D&T correction, E) Recessive model. OR: odds ratio; 95%CI: 95% confidence interval; D&T: Duval and Tweedie.
(PDF)

S2 Fig PRISMA 2009 checklist for meta-analysis. (PDF)

S3 Fig Meta-analysis of genetic association studies checklist. (DOCX)

S1 File R-script for the meta-analysis. Complete R-script for the meta-analysis of all models, plots, publication bias and sensitivity analysis.
(R) Click here for additional data file.

S1 Table NOS quality score assessment for case-control studies. (XLSX)

S2 Table List of excluded studies. (XLSX)

S3 Table Genotype and allele distribution, as well as HWE p-values for healthy controls, of all included studies. (XLSX)

S4 Table Summary of meta-analysis and Egger’s weighted regression test results for all statistical models of all analysed SNPs. (XLSX)

## References

[1] WHO | Global tuberculosis report 2014. In: WHO [Internet]. Available: http://www.who.int/tb/publications/global_report/en/. Accessed: 16 Feb 2015.

[2] Naderi M, Hashemi M, Hazire-Yazdi L, Taheri M, Moazeni-Roodi A, Eskandari-Nasab E, et al Association between toll-like receptor2 Arg677Trp and 597T/C gene polymorphisms and pulmonary tuberculosis in Zahedan, Southeast Iran. Braz J Infect Dis Off Publ Braz Soc Infect Dis. 2013;17: 516–520. 10.1016/j.bjid.2012.12.009 PMC9425122 23830055

[3] Jahantigh D, Salimi S, Alavi-Naini R, Emamdadi A, Owaysee Osquee H, Farajian Mashhadi F. Association between TLR4 and TLR9 gene polymorphisms with development of pulmonary tuberculosis in Zahedan, southeastern Iran. ScientificWorldJournal. 2013;2013: 534053 10.1155/2013/534053 23766695 PMC3677666

[4] Akira S, Uematsu S, Takeuchi O. Pathogen recognition and innate immunity. Cell. 2006;124: 783–801. 16497588 10.1016/j.cell.2006.02.015

[5] Uciechowski P, Imhoff H, Lange C, Meyer CG, Browne EN, Kirsten DK, et al Susceptibility to tuberculosis is associated with TLR1 polymorphisms resulting in a lack of TLR1 cell surface expression. J Leukoc Biol. 2011;90: 377–388. 10.1189/jlb.0409233 21642391

[6] Sha Q, Truong-Tran AQ, Plitt JR, Beck LA, Schleimer RP. Activation of airway epithelial cells by toll-like receptor agonists. Am J Respir Cell Mol Biol. 2004;31: 358–364. 10.1165/rcmb.2003-0388OC 15191912

[7] Zhang Y, Jiang T, Yang X, Xue Y, Wang C, Liu J, et al Toll-like receptor -1, -2, and -6 polymorphisms and pulmonary tuberculosis susceptibility: a systematic review and meta-analysis. PloS One. 2013;8: e63357 10.1371/journal.pone.0063357 23691034 PMC3653945

[8] Sánchez D, Lefebvre C, Rioux J, García LF, Barrera LF. Evaluation of Toll-like receptor and adaptor molecule polymorphisms for susceptibility to tuberculosis in a Colombian population. Int J Immunogenet. 2012;39: 216–223. 10.1111/j.1744-313X.2011.01077.x 22221660

[9] Xue Y, Zhao ZQ, Wang HJ, Jin L, Liu CP, Wang Y, et al Toll-like receptors 2 and 4 gene polymorphisms in a southeastern Chinese population with tuberculosis. Int J Immunogenet. 2010;37: 135–138. 10.1111/j.1744-313X.2009.00892.x 20002809

[10] Yamamoto M, Sato S, Hemmi H, Hoshino K, Kaisho T, Sanjo H, et al Role of adaptor TRIF in the MyD88-independent toll-like receptor signaling pathway. Science. 2003;301: 640–643. 10.1126/science.1087262 12855817

[11] Najmi N, Kaur G, Sharma SK, Mehra NK. Human Toll-like receptor 4 polymorphisms TLR4 Asp299Gly and Thr399Ile influence susceptibility and severity of pulmonary tuberculosis in the Asian Indian population. Tissue Antigens. 2010;76: 102–109. 10.1111/j.1399-0039.2010.01481.x 20403143

[12] Newport MJ, Allen A, Awomoyi AA, Dunstan SJ, McKinney E, Marchant A, et al The toll-like receptor 4 Asp299Gly variant: no influence on LPS responsiveness or susceptibility to pulmonary tuberculosis in The Gambia. Tuberc Edinb Scotl. 2004;84: 347–352. 10.1016/j.tube.2004.02.001 15525557

[13] Lyman GH, Kuderer NM. The strengths and limitations of meta-analyses based on aggregate data. BMC Med Res Methodol. 2005;5: 14 10.1186/1471-2288-5-14 15850485 PMC1097735

[14] Meta-analysis: Its strengths and limitations : Cleveland Clinic Journal of Medicine [Internet]. Available: http://www.ccjm.org/index.php?id=107953&cHash=010515&tx_ttnews%5Btt_news%5D=360745. Accessed 18 Feb 2015.

[15] Readings_The Newcastle—Scale for assessing the quality of nonrandomised studies in meta-analyses.pdf.

[16] Higgins JPT, Thompson SG. Quantifying heterogeneity in a meta-analysis. Stat Med. 2002;21: 1539–1558. 10.1002/sim.1186 12111919

[17] Viechtbauer W, others. Conducting meta-analyses in R with the metafor package. J Stat Softw. 2010;36: 1–48.

[18] Peters JL, Sutton AJ, Jones DR, Abrams KR, Rushton L. Comparison of two methods to detect publication bias in meta-analysis. JAMA. 2006;295: 676–680. 10.1001/jama.295.6.676 16467236

[19] Duval S, Tweedie R. Trim and fill: A simple funnel-plot-based method of testing and adjusting for publication bias in meta-analysis. Biometrics. 2000;56: 455–463. 10877304 10.1111/j.0006-341x.2000.00455.x

[20] Sinha E, Biswas SK, Mittal M, Bajaj B, Singh V, Arela N, et al Toll-like Receptor 1 743 A>G, 1805 T>G & Toll-like Receptor 6 745 C>T gene polymorphism and tuberculosis: a case control study of north Indian population from Agra (India). Hum Immunol. 2014;75: 880–886. 24984237 10.1016/j.humimm.2014.06.014

[21] Qi H, Sun L, Wu X, Jin Y, Xiao J, Wang S, et al Toll-like receptor 1(TLR1) Gene SNP rs5743618 is associated with increased risk for tuberculosis in Han Chinese children. Tuberc Edinb Scotl. 2015;95: 197–203. 10.1016/j.tube.2014.12.001 25544311

[22] Dittrich N, Berrocal-Almanza LC, Thada S, Goyal S, Slevogt H, Sumanlatha G, et al Toll-like receptor 1 variations influence susceptibility and immune response to Mycobacterium. Tuberculosis. 0 10.1016/j.tube.2015.02.045 25857934

[23] Salie M, Daya M, Lucas LA, Warren RM, van der Spuy GD, van Helden PD, et al Association of toll-like receptors with susceptibility to tuberculosis suggests sex-specific effects of TLR8 polymorphisms. Infect Genet Evol J Mol Epidemiol Evol Genet Infect Dis. 2015; 10.1016/j.meegid.2015.07.004 26160538

[24] Ma X, Liu Y, Gowen BB, Graviss EA, Clark AG, Musser JM. Full-Exon Resequencing Reveals Toll-Like Receptor Variants Contribute to Human Susceptibility to Tuberculosis Disease. PLoS ONE. 2007;2 10.1371/journal.pone.0001318 PMC2117342 18091991

[25] Ma M, Xie L, Wu S, Tang F, Li H, Zhang Z, et al Toll-like receptors, tumor necrosis factor-α, and interleukin–10 gene polymorphisms in risk of pulmonary tuberculosis and disease severity. Hum Immunol. 2010;71: 1005–1010. 20650298 10.1016/j.humimm.2010.07.009

[26] Selvaraj P, Harishankar M, Singh B, Jawahar MS, Banurekha VV. Toll-like receptor and TIRAP gene polymorphisms in pulmonary tuberculosis patients of South India. Tuberc Edinb Scotl. 2010;90: 306–310. 10.1016/j.tube.2010.08.001 20797905

[27] Wu L, Hu Y, Li D, Jiang W, Xu B. Screening toll-like receptor markers to predict latent tuberculosis infection and subsequent tuberculosis disease in a Chinese population. BMC Med Genet. 2015;16: 19 10.1186/s12881-015-0166-1 25928077 PMC4421918

[28] Ocejo-Vinyals JG, Puente de Mateo E, Ausín F, Agüero R, Arroyo JL, Gutiérrez-Cuadra M, et al Human toll-like receptor 1 T1805G polymorphism and susceptibility to pulmonary tuberculosis in northern Spain. Int J Tuberc Lung Dis Off J Int Union Tuberc Lung Dis. 2013;17: 652–654. 10.5588/ijtld.12.0767 23575331

[29] Caws M, Thwaites G, Dunstan S, Hawn TR, Lan NTN, Thuong NTT, et al The influence of host and bacterial genotype on the development of disseminated disease with Mycobacterium tuberculosis. PLoS Pathog. 2008;4: e1000034 10.1371/journal.ppat.1000034 18369480 PMC2268004

[30] Yang Y, Li X, Cui W, Guan L, Shen F, Xu J, et al Potential association of pulmonary tuberculosis with genetic polymorphisms of toll-like receptor 9 and interferon-gamma in a Chinese population. BMC Infect Dis. 2013;13: 511 10.1186/1471-2334-13-511 24176007 PMC3819710

[31] Arji N, Busson M, Iraqi G, Bourkadi JE, Benjouad A, Bouayad A, et al Genetic diversity of TLR2, TLR4, and VDR loci and pulmonary tuberculosis in Moroccan patients. J Infect Dev Ctries. 2014;8: 430–440. 10.3855/jidc.3820 24727508

[32] Etokebe GE, Skjeldal F, Nilsen N, Rodionov D, Knezevic J, Bulat-Kardum L, et al Toll-like receptor 2 (P631H) mutant impairs membrane internalization and is a dominant negative allele. Scand J Immunol. 2010;71: 369–381. 10.1111/j.1365-3083.2010.02379.x 20500688

[33] Torres-García D, Cruz-Lagunas A, García-Sancho Figueroa MC, Fernández-Plata R, Baez-Saldaña R, Mendoza-Milla C, et al Variants in toll-like receptor 9 gene influence susceptibility to tuberculosis in a Mexican population. J Transl Med. 2013;11: 220 10.1186/1479-5876-11-220 24053111 PMC3849691

[34] Xue Y, Jin L, Li A-Z, Wang H-J, Li M, Zhang Y-X, et al Microsatellite polymorphisms in intron 2 of the toll-like receptor 2 gene and their association with susceptibility to pulmonary tuberculosis in Han Chinese. Clin Chem Lab Med CCLM FESCC. 2010;48: 785–789. 10.1515/CCLM.2010.154 20298136

[35] Dalgic N, Tekin D, Kayaalti Z, Soylemezoglu T, Cakir E, Kilic B, et al Arg753Gln polymorphism of the human Toll-like receptor 2 gene from infection to disease in pediatric tuberculosis. Hum Immunol. 2011;72: 440–445. 10.1016/j.humimm.2011.02.001 21320563

[36] Chen Y-C, Hsiao C-C, Chen C-J, Chin C-H, Liu S-F, Wu C-C, et al Toll-like receptor 2 gene polymorphisms, pulmonary tuberculosis, and natural killer cell counts. BMC Med Genet. 2010;11: 17 10.1186/1471-2350-11-17 20113509 PMC2824655

[37] Yim J-J, Lee HW, Lee HS, Kim YW, Han SK, Shim Y-S, et al The association between microsatellite polymorphisms in intron II of the human Toll-like receptor 2 gene and tuberculosis among Koreans. Genes Immun. 2006;7: 150–155. 10.1038/sj.gene.6364274 16437124

[38] Fitness J, Floyd S, Warndorff DK, Sichali L, Malema S, Crampin AC, et al Large-scale candidate gene study of tuberculosis susceptibility in the Karonga district of northern Malawi. Am J Trop Med Hyg. 2004;71: 341–349. 15381817

[39] Olesen R, Wejse C, Velez DR, Bisseye C, Sodemann M, Aaby P, et al DC-SIGN (CD209), pentraxin 3 and vitamin D receptor gene variants associate with pulmonary tuberculosis risk in West Africans. Genes Immun. 2007;8: 456–467. 10.1038/sj.gene.6364410 17611589

[40] Rosas-Taraco AG, Revol A, Salinas-Carmona MC, Rendon A, Caballero-Olin G, Arce-Mendoza AY. CD14 C(-159)T polymorphism is a risk factor for development of pulmonary tuberculosis. J Infect Dis. 2007;196: 1698–1706. 10.1086/522147 18008256

[41] Davila S, Hibberd ML, Hari Dass R, Wong HEE, Sahiratmadja E, Bonnard C, et al Genetic association and expression studies indicate a role of toll-like receptor 8 in pulmonary tuberculosis. PLoS Genet. 2008;4: e1000218 10.1371/journal.pgen.1000218 18927625 PMC2568981

[42] Dalgic N, Tekin D, Kayaalti Z, Cakir E, Soylemezoglu T, Sancar M. Relationship between toll-like receptor 8 gene polymorphisms and pediatric pulmonary tuberculosis. Dis Markers. 2011;31: 33–38. 10.3233/DMA-2011-0800 21846947 PMC3826908

[43] Hashemi-Shahri SM, Taheri M, Gadari A, Naderi M, Bahari G, Hashemi M. Association Between TLR8 and TLR9 Gene Polymorphisms and Pulmonary Tuberculosis. Gene Cell Tissue. 2014;1 Available: http://genecelltissue.com/18316.abstract.

[44] Bukhari M, Aslam MA, Khan A, Iram Q, Akbar A, Naz AG, et al TLR8 gene polymorphism and association in bacterial load in southern Punjab of Pakistan: an association study with pulmonary tuberculosis. Int J Immunogenet. 2015;42: 46–51. 10.1111/iji.12170 25572425

[45] Kobayashi K, Yuliwulandari R, Yanai H, Naka I, Lien LT, Hang NTL, et al Association of TLR polymorphisms with development of tuberculosis in Indonesian females. Tissue Antigens. 2012;79: 190–197. 10.1111/j.1399-0039.2011.01821.x 22211722

[46] Hawn TR, Misch EA, Dunstan SJ, Thwaites GE, Lan NTN, Quy HT, et al A common human TLR1 polymorphism regulates the innate immune response to lipopeptides. Eur J Immunol. 2007;37: 2280–2289. 10.1002/eji.200737034 17595679

[47] Ogus AC, Yoldas B, Ozdemir T, Uguz A, Olcen S, Keser I, et al The Arg753GLn polymorphism of the human toll-like receptor 2 gene in tuberculosis disease. Eur Respir J. 2004;23: 219–223. 14979495 10.1183/09031936.03.00061703

[48] Schröder NWJ, Diterich I, Zinke A, Eckert J, Draing C, von Baehr V, et al Heterozygous Arg753Gln polymorphism of human TLR–2 impairs immune activation by Borrelia burgdorferi and protects from late stage Lyme disease. J Immunol Baltim Md 1950. 2005;175: 2534–2540. 10.4049/jimmunol.175.4.2534 16081826

[49] Cavalcanti YVN, Brelaz MCA, Neves JK de AL, Candido Ferraz J, Pereira V, et al Role of TNF-Alpha, IFN-Gamma, and IL–10 in the Development of Pulmonary Tuberculosis, Role of TNF-Alpha, IFN-Gamma, and IL–10 in the Development of Pulmonary Tuberculosis. Pulm Med Pulm Med. 2012;2012, 2012: e745483 10.1155/2012/745483 PMC3515941 23251798

[50] Sun Q, Zhang Q, Xiao H-P, Bai C. Toll-like receptor polymorphisms and tuberculosis susceptibility: A comprehensive meta-analysis. J Huazhong Univ Sci Technol Med Sci Hua Zhong Ke Ji Xue Xue Bao Yi Xue Ying Wen Ban Huazhong Keji Daxue Xuebao Yixue Yingdewen Ban. 2015;35: 157–168. 10.1007/s11596-015-1405-6 25877346

[51] Wang J-J, Xia X, Tang S-D, Wang J, Deng X-Z, Zhang Y, et al Meta-Analysis on the Associations of TLR2 Gene Polymorphisms with Pulmonary Tuberculosis Susceptibility among Asian Populations. PLoS ONE. 2013;8: e75090 10.1371/journal.pone.0075090 24124467 PMC3790778

[52] Tian T, Jin S, Dong J, Li G. Lack of association between Toll-like receptor 4 gene Asp299Gly and Thr399Ile polymorphisms and tuberculosis susceptibility: a meta-analysis. Infect Genet Evol J Mol Epidemiol Evol Genet Infect Dis. 2013;14: 156–160. 10.1016/j.meegid.2012.11.009 23200920

[53] Shey MS, Randhawa AK, Bowmaker M, Smith E, Scriba TJ, de Kock M, et al Single nucleotide polymorphisms in toll-like receptor 6 are associated with altered lipopeptide- and mycobacteria-induced interleukin–6 secretion. Genes Immun. 2010;11: 561–572. 10.1038/gene.2010.14 20445564 PMC3518443

[54] Randhawa AK, Shey MS, Keyser A, Peixoto B, Wells RD, de Kock M, et al Association of Human TLR1 and TLR6 Deficiency with Altered Immune Responses to BCG Vaccination in South African Infants. PLoS Pathog. 2011;7 10.1371/journal.ppat.1002174 PMC3154845 21852947

[55] Huang L-Y, Ishii KJ, Akira S, Aliberti J, Golding B. Th1-like cytokine induction by heat-killed Brucella abortus is dependent on triggering of TLR9. J Immunol Baltim Md 1950. 2005;175: 3964–3970. 10.4049/jimmunol.175.6.3964 16148144

[56] Tao K, Fujii M, Tsukumo S, Maekawa Y, Kishihara K, Kimoto Y, et al Genetic variations of Toll-like receptor 9 predispose to systemic lupus erythematosus in Japanese population. Ann Rheum Dis. 2007;66: 905–909. 10.1136/ard.2006.065961 17344245 PMC1955115

[57] Zhao L, Liu K, Kong X, Tao Z, Wang Y, Liu Y. Association of polymorphisms in Toll-like receptors 4 and 9 with risk of pulmonary tuberculosis: a meta-analysis. Med Sci Monit Int Med J Exp Clin Res. 2015;21: 1097–1106. 10.12659/MSM.893755 PMC4412087 25889916

[58] Chen Z, Wang W, Liang J, Wang J, Feng S, Zhang G. Association between toll-like receptors 9 (TLR9) gene polymorphism and risk of pulmonary tuberculosis: meta-analysis. BMC Pulm Med. 2015;15: 57 10.1186/s12890-015-0049-4 25948535 PMC4460768

[59] Thada S, Valluri VL, Gaddam SL. Influence of Toll-like receptor gene polymorphisms to tuberculosis susceptibility in humans. Scand J Immunol. 2013;78: 221–229. 10.1111/sji.12066 23672492

[60] Sultani M, Stringer AM, Bowen JM, Gibson RJ, Sultani M, Stringer AM, et al Anti-Inflammatory Cytokines: Important Immunoregulatory Factors Contributing to Chemotherapy-Induced Gastrointestinal Mucositis, Anti-Inflammatory Cytokines: Important Immunoregulatory Factors Contributing to Chemotherapy-Induced Gastrointestinal Mucositis. Chemother Res Pract Chemother Res Pract. 2012;2012, 2012: e490804 10.1155/2012/490804 PMC3437608 22973511

[61] Daya M, van der Merwe L, van Helden PD, Möller M, Hoal EG. The role of ancestry in TB susceptibility of an admixed South African population. Tuberc Edinb Scotl. 2014;94: 413–420. 10.1016/j.tube.2014.03.012 24832562

[62] Ensembl genome browser 81: Homo sapiens—Population genetics—rs5743708 (SNP) [Internet]. [cited 3 Sep 2015]. Available: http://www.ensembl.org/Homo_sapiens/Variation/Population?db=core;r=4:153704665-153705665;v=rs5743708;vdb=variation;vf=3040389#population_freq_AMR.

[63] Sherry ST, Ward MH, Kholodov M, Baker J, Phan L, Smigielski EM, et al dbSNP: the NCBI database of genetic variation. Nucleic Acids Res. 2001;29: 308–311. 11125122 10.1093/nar/29.1.308 PMC29783

[64] Thye T, Nejentsev S, Intemann CD, Browne EN, Chinbuah MA, Gyapong J, et al MCP–1 promoter variant -362C associated with protection from pulmonary tuberculosis in Ghana, West Africa. Hum Mol Genet. 2009;18: 381–388. 10.1093/hmg/ddn352 18940815 PMC2638774

[65] Di Pietrantonio T, Schurr E. Host-pathogen specificity in tuberculosis. Adv Exp Med Biol. 2013;783: 33–44. 10.1007/978-1-4614-6111-1_2 23468102

[66] Cordell HJ, Clayton DG. Genetic association studies. Lancet. 2005;366: 1121–1131. 10.1016/S0140-6736(05)67424-7 16182901

[67] Hawn TR, Shah JA, Kalman D. New tricks for old dogs: countering antibiotic resistance in tuberculosis with host-directed therapeutics. Immunol Rev. 2015;264: 344–362. 10.1111/imr.12255 25703571 PMC4571192

[68] Tobin DM, Roca FJ, Oh SF, McFarland R, Vickery TW, Ray JP, et al Host genotype-specific therapies can optimize the inflammatory response to mycobacterial infections. Cell. 2012;148: 434–446. 10.1016/j.cell.2011.12.023 22304914 PMC3433720

[69] Thye T, Vannberg FO, Wong SH, Owusu-Dabo E, Osei I, Gyapong J, et al Genome-wide association analyses identifies a susceptibility locus for tuberculosis on chromosome 18q11.2. Nat Genet. 2010;42: 739–741. 10.1038/ng.639 20694014 PMC4975513

[70] Chimusa ER, Zaitlen N, Daya M, Möller M, van Helden PD, Mulder NJ, et al Genome-wide association study of ancestry-specific TB risk in the South African Coloured population. Hum Mol Genet. 2014;23: 796–809. 10.1093/hmg/ddt462 24057671 PMC3888262

[71] Png E, Alisjahbana B, Sahiratmadja E, Marzuki S, Nelwan R, Balabanova Y, et al A genome wide association study of pulmonary tuberculosis susceptibility in Indonesians. BMC Med Genet. 2012;13: 5 10.1186/1471-2350-13-5 22239941 PMC3287960

[72] Ji L-D, Chai P-F, Zhou B-B, Tang NLS, Xing W-H, Yuan F, et al Lack of association between polymorphisms from genome-wide association studies and tuberculosis in the Chinese population. Scand J Infect Dis. 2013;45: 310–314. 10.3109/00365548.2012.726739 23113532

