# Supplementary material for: TLR1, 2, 4, 6 and 9 Variants Associated with Tuberculosis Susceptibility: A Systematic Review and Meta-Analysis
Source: PLoS One. 2015 Oct 2;10(10):e0139711. doi: 10.1371/journal.pone.0139711 (PMC4592262; doi:10.1371/journal.pone.0139711)

TLR1 rs5743618

A)

Author(s) and Year	Case Events	Case Total	Control Events	Control Total	Weight	OR [95% CI]
Sinha_et_al., 2014	19	320	26	248	10.03%	0.54 [0.29 , 1.00]
Ma_et_al., 2010	34	1086	36	1122	11.18%	0.97 [0.61 , 1.57]
Selvaraj_et_al., 2010	11	412	17	424	8.77%	0.66 [0.30 , 1.42]
Ma_et_al., 2007	71	678	87	388	12.18%	0.40 [0.29 , 0.57]
Ocejo−Vinyals_et_al., 2013	198	380	166	384	12.55%	1.43 [1.07 , 1.90]
Ma_et_al., 2007	123	750	67	228	12.17%	0.47 [0.33 , 0.67]
Qi_et_al., 2015	45	680	21	732	10.76%	2.40 [1.41 , 4.07]
Salie_et_al., 2015	96	656	93	660	12.41%	1.05 [0.77 , 1.42]
Wu_et_al., 2015	12	218	74	844	9.93%	0.61 [0.32 , 1.14]
RE Model					100.00%	0.81 [0.55 , 1.19]

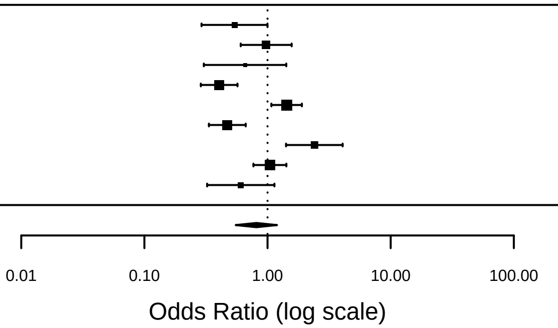

Ai)

Author(s) and Year	Case Events	Case Total	Control Events	Control Total	Weight	OR [95% CI]
Sinha_et_al., 2014	19	320	26	248	9.07%	0.54 [0.29 , 1.00]
Ma_et_al., 2010	34	1086	36	1122	9.99%	0.97 [0.61 , 1.57]
Selvaraj_et_al., 2010	11	412	17	424	8.04%	0.66 [0.30 , 1.42]
Ma_et_al., 2007	71	678	87	388	10.76%	0.40 [0.29 , 0.57]
Ocejo−Vinyals_et_al., 2013	198	380	166	384	11.05%	1.43 [1.07 , 1.90]
Ma_et_al., 2007	123	750	67	228	10.75%	0.47 [0.33 , 0.67]
Qi_et_al., 2015	45	680	21	732	9.65%	2.40 [1.41 , 4.07]
Salie_et_al., 2015	96	656	93	660	10.94%	1.05 [0.77 , 1.42]
Wu_et_al., 2015	12	218	74	844	8.99%	0.61 [0.32 , 1.14]
D&T_Filler, 2015	0	0	0	0	10.76%	1.98 [1.41 , 2.79]
RE Model					100.00%	0.89 [0.61 , 1.32]

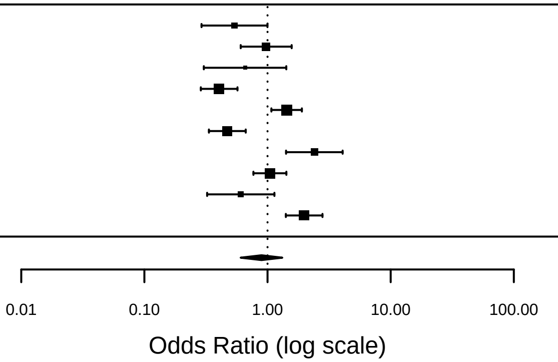

B)

Author(s) and Year	Case Events	Case Total	Control Events	Control Total	Weight	OR [95% CI]
Sinha_et_al., 2014	0	141	1	100	5.34%	0.23 [0.01 , 5.81]
Ma_et_al., 2010	1	511	1	527	6.62%	1.03 [0.06 , 16.53]
Selvaraj_et_al., 2010	1	197	0	195	5.35%	2.98 [0.12 , 73.72]
Ma_et_al., 2007	4	276	13	133	16.17%	0.14 [0.04 , 0.42]
Ocejo−Vinyals_et_al., 2013	58	108	34	94	20.77%	2.05 [1.16 , 3.60]
Ma_et_al., 2007	20	292	14	75	19.51%	0.32 [0.15 , 0.67]
Qi_et_al., 2015	0	295	0	345	3.87%	1.17 [0.02 , 59.11]
Salie_et_al., 2015	3	238	7	251	14.36%	0.44 [0.11 , 1.74]
Wu_et_al., 2015	1	99	2	352	8.02%	1.79 [0.16 , 19.90]
RE Model					100.00%	0.62 [0.27 , 1.45]

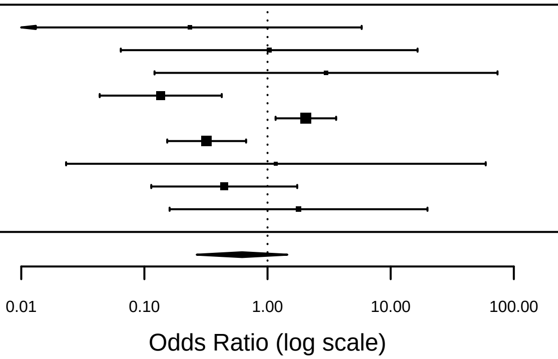

C)

Author(s) and Year	Case Events	Case Total	Control Events	Control Total	Weight	OR [95% CI]
Sinha_et_al., 2014	19	160	99	123	10.12%	0.56 [0.29 , 1.07]
Ma_et_al., 2010	32	542	526	560	11.59%	0.97 [0.59 , 1.60]
Selvaraj_et_al., 2010	9	205	195	212	8.53%	0.53 [0.23 , 1.21]
Ma_et_al., 2007	63	335	120	181	12.36%	0.46 [0.30 , 0.69]
Ocejo−Vinyals_et_al., 2013	82	132	60	158	11.79%	1.00 [0.62 , 1.62]
Ma_et_al., 2007	83	355	61	100	11.84%	0.48 [0.30 , 0.76]
Qi_et_al., 2015	45	340	345	366	11.19%	2.51 [1.46 , 4.30]
Salie_et_al., 2015	90	325	244	323	12.88%	1.18 [0.83 , 1.68]
Wu_et_al., 2015	10	108	350	420	9.70%	0.51 [0.25 , 1.03]
RE Model					100.00%	0.78 [0.54 , 1.15]

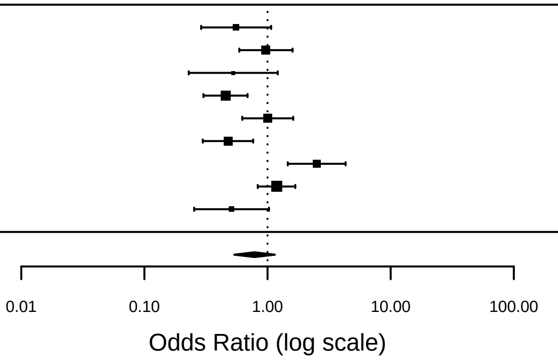

TLR1 rs5743618

Ci)

Author(s) and Year	Case Events	Case Total	Control Events	Control Total	Weight	OR [95% CI]
Sinha_et_al., 2014	19	160	99	123	8.29%	0.56 [0.29 , 1.07]
Ma_et_al., 2010	32	542	526	560	9.31%	0.97 [0.59 , 1.60]
Selvaraj_et_al., 2010	9	205	195	212	7.15%	0.53 [0.23 , 1.21]
Ma_et_al., 2007	63	335	120	181	9.83%	0.46 [0.30 , 0.69]
Ocejo−Vinyals_et_al., 2013	82	132	60	158	9.44%	1.00 [0.62 , 1.62]
Ma_et_al., 2007	83	355	61	100	9.48%	0.48 [0.30 , 0.76]
Qi_et_al., 2015	45	340	345	366	9.03%	2.51 [1.46 , 4.30]
Salie_et_al., 2015	90	325	244	323	10.17%	1.18 [0.83 , 1.68]
Wu_et_al., 2015	10	108	350	420	8.00%	0.51 [0.25 , 1.03]
D&T_Filler, 2015	0	0	0	0	9.48%	1.82 [1.13 , 2.91]
D&T_Filler, 2015	0	0	0	0	9.83%	1.90 [1.26 , 2.87]
RE Model					100.00%	0.92 [0.64 , 1.34]

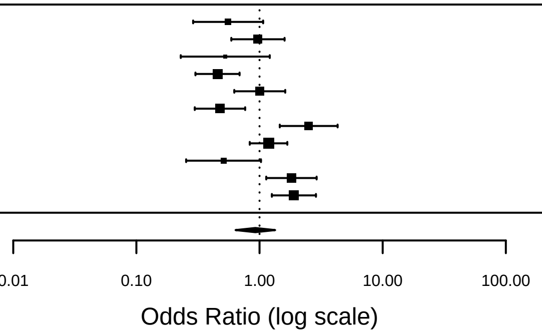

D)

Author(s) and Year	Case Events	Case Total	Control Events	Control Total	Weight	OR [95% CI]
Sinha_et_al., 2014	141	160	99	124	10.16%	0.53 [0.28 , 1.02]
Ma_et_al., 2010	510	543	526	561	11.46%	0.97 [0.60 , 1.59]
Selvaraj_et_al., 2010	196	206	195	212	8.90%	0.59 [0.26 , 1.31]
Ma_et_al., 2007	272	339	120	194	12.19%	0.40 [0.27 , 0.59]
Ocejo−Vinyals_et_al., 2013	50	190	60	192	11.82%	1.27 [0.82 , 1.98]
Ma_et_al., 2007	272	375	61	114	11.91%	0.44 [0.28 , 0.67]
Qi_et_al., 2015	295	340	345	366	11.06%	2.51 [1.46 , 4.30]
Salie_et_al., 2015	235	328	244	330	12.54%	1.12 [0.80 , 1.58]
Wu_et_al., 2015	98	109	350	422	9.97%	0.55 [0.28 , 1.07]
RE Model					100.00%	0.79 [0.52 , 1.18]

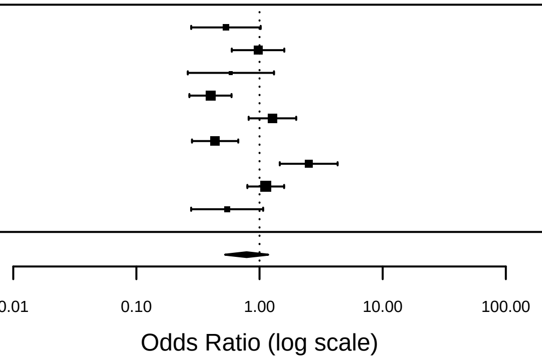

Di)

Author(s) and Year	Case Events	Case Total	Control Events	Control Total	Weight	OR [95% CI]
Sinha_et_al., 2014	141	160	99	124	8.40%	0.53 [0.28 , 1.02]
Ma_et_al., 2010	510	543	526	561	9.21%	0.97 [0.60 , 1.59]
Selvaraj_et_al., 2010	196	206	195	212	7.56%	0.59 [0.26 , 1.31]
Ma_et_al., 2007	272	339	120	194	9.66%	0.40 [0.27 , 0.59]
Ocejo−Vinyals_et_al., 2013	50	190	60	192	9.43%	1.27 [0.82 , 1.98]
Ma_et_al., 2007	272	375	61	114	9.49%	0.44 [0.28 , 0.67]
Qi_et_al., 2015	295	340	345	366	8.97%	2.51 [1.46 , 4.30]
Salie_et_al., 2015	235	328	244	330	9.86%	1.12 [0.80 , 1.58]
Wu_et_al., 2015	98	109	350	422	8.27%	0.55 [0.28 , 1.07]
D&T_Filler, 2015	0	0	0	0	9.49%	2.16 [1.40 , 3.33]
D&T_Filler, 2015	0	0	0	0	9.66%	2.36 [1.59 , 3.49]
RE Model					100.00%	0.96 [0.63 , 1.45]

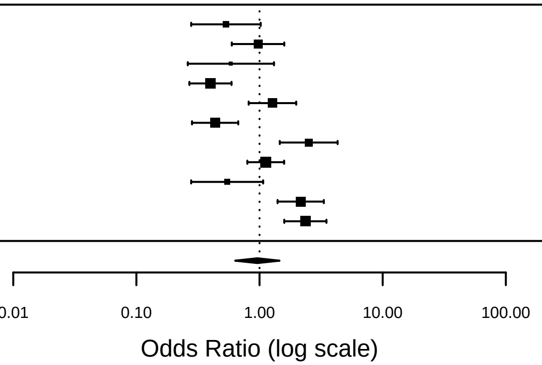

E)

Author(s) and Year	Case Events	Case Total	Control Events	Control Total	Weight	OR [95% CI]
Sinha_et_al., 2014	0	160	1	124	4.96%	0.26 [0.01 , 6.35]
Ma_et_al., 2010	1	543	1	561	6.21%	1.03 [0.06 , 16.56]
Selvaraj_et_al., 2010	1	206	0	212	4.97%	3.10 [0.13 , 76.59]
Ma_et_al., 2007	4	339	13	194	16.23%	0.17 [0.05 , 0.52]
Ocejo−Vinyals_et_al., 2013	58	190	34	192	22.11%	2.04 [1.26 , 3.31]
Ma_et_al., 2007	20	375	14	114	20.15%	0.40 [0.20 , 0.83]
Qi_et_al., 2015	0	340	0	366	3.57%	1.08 [0.02 , 54.40]
Salie_et_al., 2015	3	328	7	330	14.21%	0.43 [0.11 , 1.66]
Wu_et_al., 2015	1	109	2	422	7.59%	1.94 [0.17 , 21.64]
RE Model					100.00%	0.67 [0.30 , 1.50]

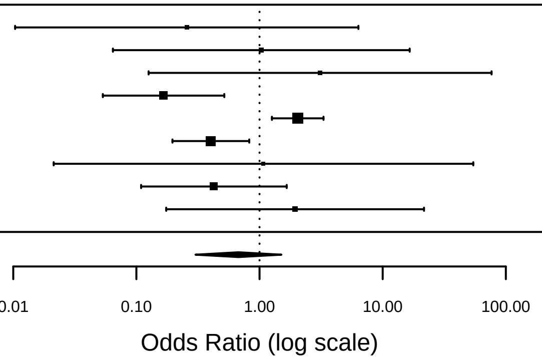

TLR1 rs5743618 Asian

A)

Author(s) and Year	Case Events	Case Total	Control Events	Control Total	Weight	OR [95% CI]
Sinha_et_al., 2014	19	320	26	248	19.81%	0.54 [0.29 , 1.00]
Ma_et_al., 2010	34	1086	36	1122	22.01%	0.97 [0.61 , 1.57]
Selvaraj_et_al., 2010	11	412	17	424	17.37%	0.66 [0.30 , 1.42]
Qi_et_al., 2015	45	680	21	732	21.20%	2.40 [1.41 , 4.07]
Wu_et_al., 2015	12	218	74	844	19.61%	0.61 [0.32 , 1.14]
RE Model					100.00%	0.89 [0.51 , 1.55]

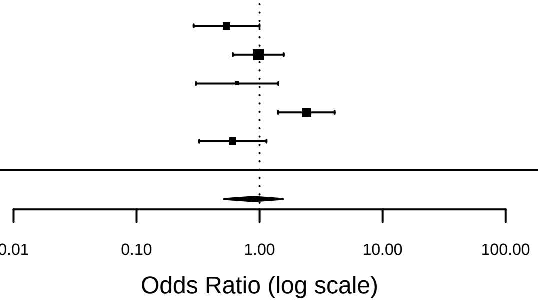

Ai)

Author(s) and Year	Case Events	Case Total	Control Events	Control Total	Weight	OR [95% CI]
Sinha_et_al., 2014	19	320	26	248	16.54%	0.54 [0.29 , 1.00]
Ma_et_al., 2010	34	1086	36	1122	18.34%	0.97 [0.61 , 1.57]
Selvaraj_et_al., 2010	11	412	17	424	14.54%	0.66 [0.30 , 1.42]
Qi_et_al., 2015	45	680	21	732	17.67%	2.40 [1.41 , 4.07]
Wu_et_al., 2015	12	218	74	844	16.37%	0.61 [0.32 , 1.14]
D&T_Filler, 2015	0	0	0	0	16.54%	1.89 [1.02 , 3.50]
RE Model					100.00%	1.01 [0.61 , 1.68]

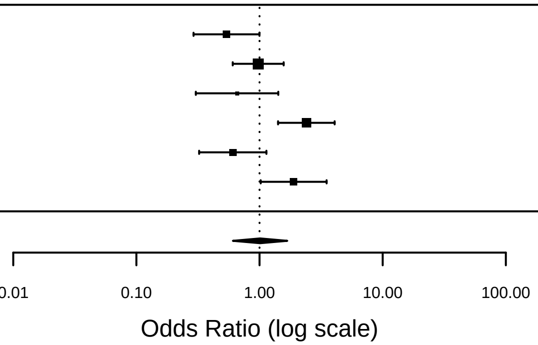

B)

Author(s) and Year	Case Events	Case Total	Control Events	Control Total	Weight	OR [95% CI]
Sinha_et_al., 2014	0	141	1	100	17.28%	0.23 [0.01 , 5.81]
Ma_et_al., 2010	1	511	1	527	23.15%	1.03 [0.06 , 16.53]
Selvaraj_et_al., 2010	1	197	0	195	17.33%	2.98 [0.12 , 73.72]
Qi_et_al., 2015	0	295	0	345	11.58%	1.17 [0.02 , 59.11]
Wu_et_al., 2015	1	99	2	352	30.66%	1.79 [0.16 , 19.90]
FE Model					100.00%	1.15 [0.30 , 4.38]

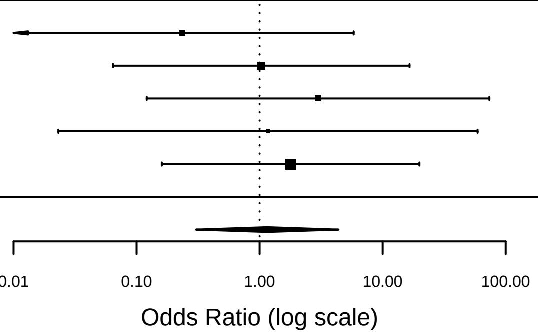

C)

Author(s) and Year	Case Events	Case Total	Control Events	Control Total	Weight	OR [95% CI]
Sinha_et_al., 2014	19	160	99	123	19.88%	0.56 [0.29 , 1.07]
Ma_et_al., 2010	32	542	526	560	21.98%	0.97 [0.59 , 1.60]
Selvaraj_et_al., 2010	9	205	195	212	17.45%	0.53 [0.23 , 1.21]
Qi_et_al., 2015	45	340	345	366	21.42%	2.51 [1.46 , 4.30]
Wu_et_al., 2015	10	108	350	420	19.26%	0.51 [0.25 , 1.03]
RE Model					100.00%	0.85 [0.46 , 1.56]

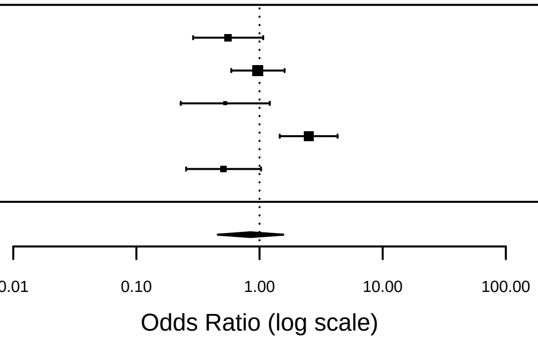

TLR1 rs5743618 Asian

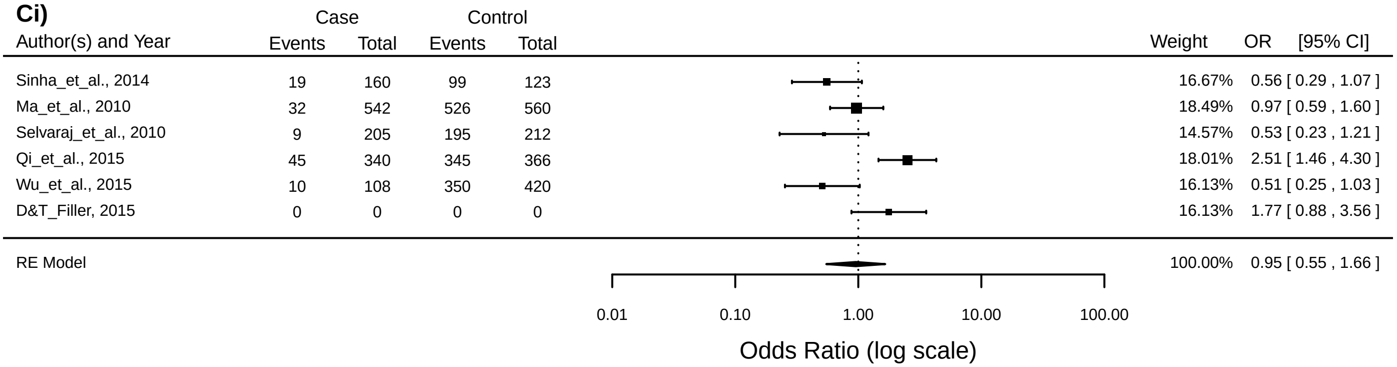

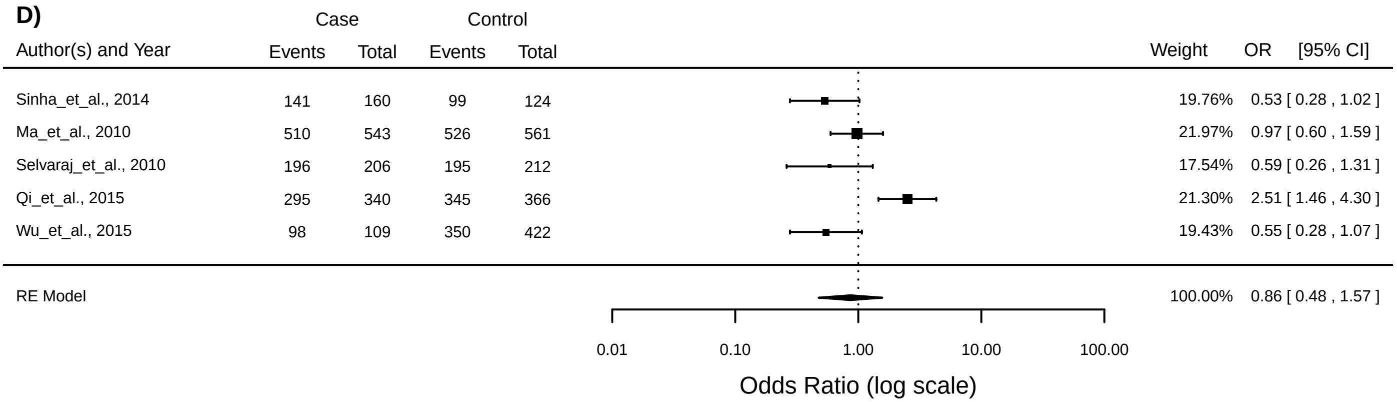

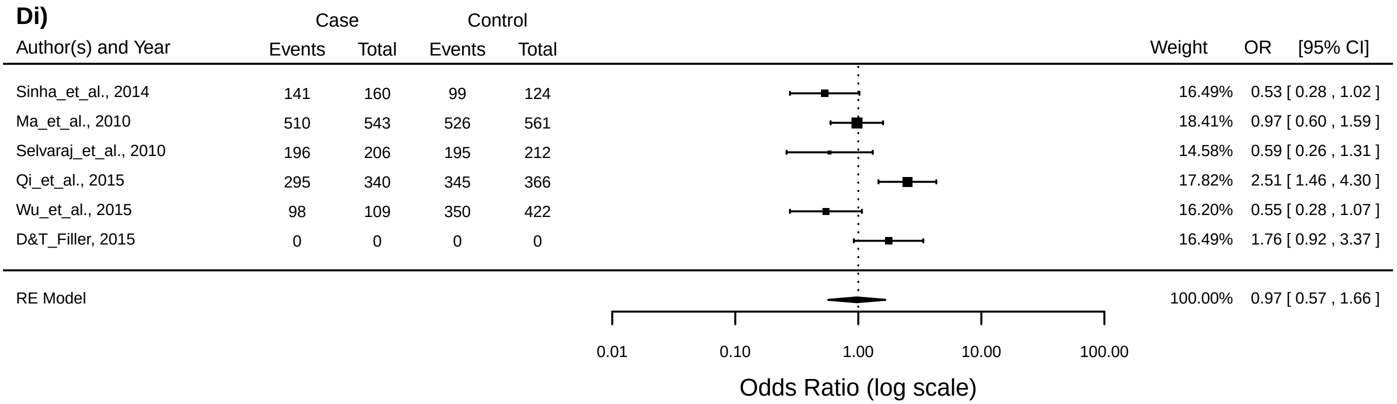

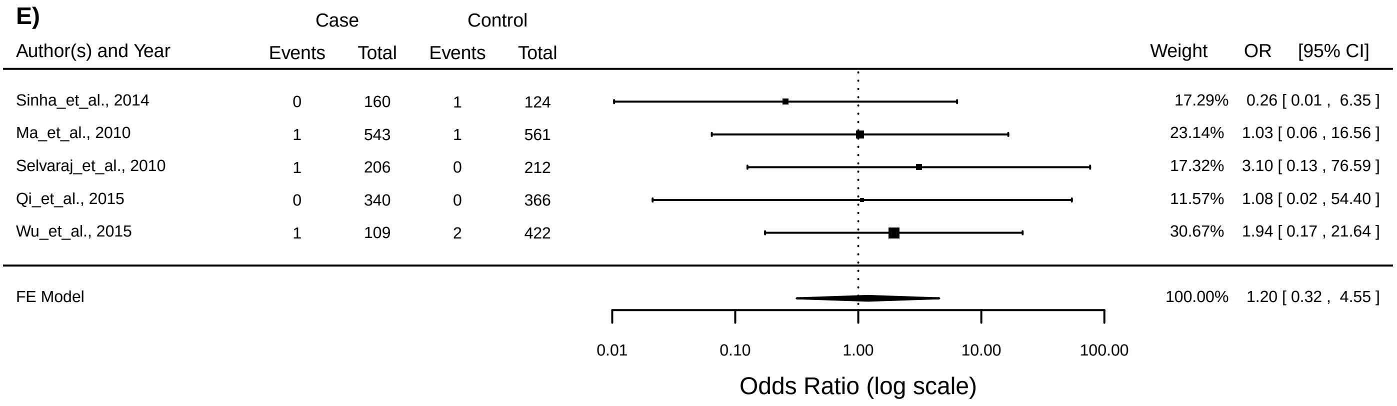

TLR2 rs3804099

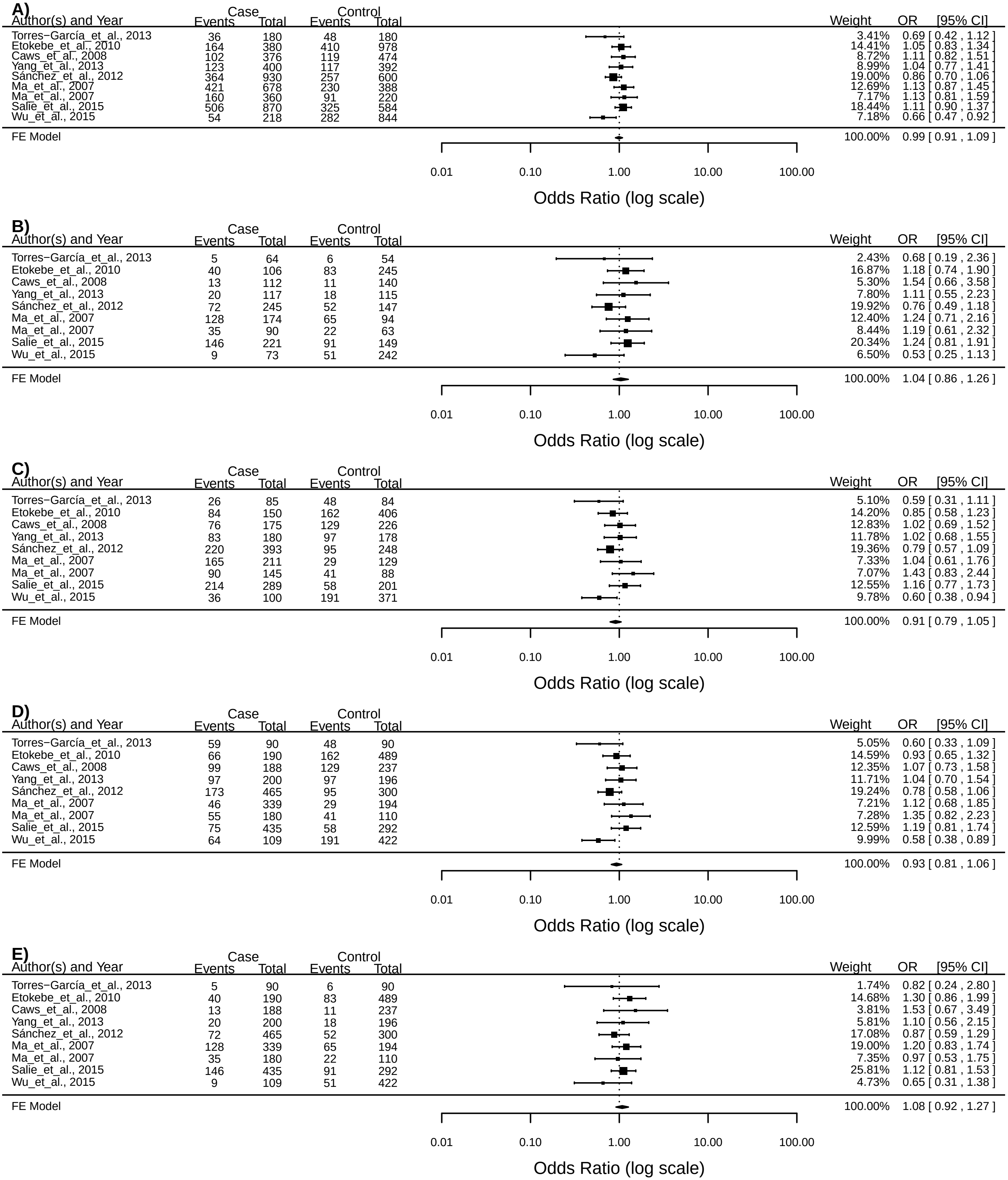

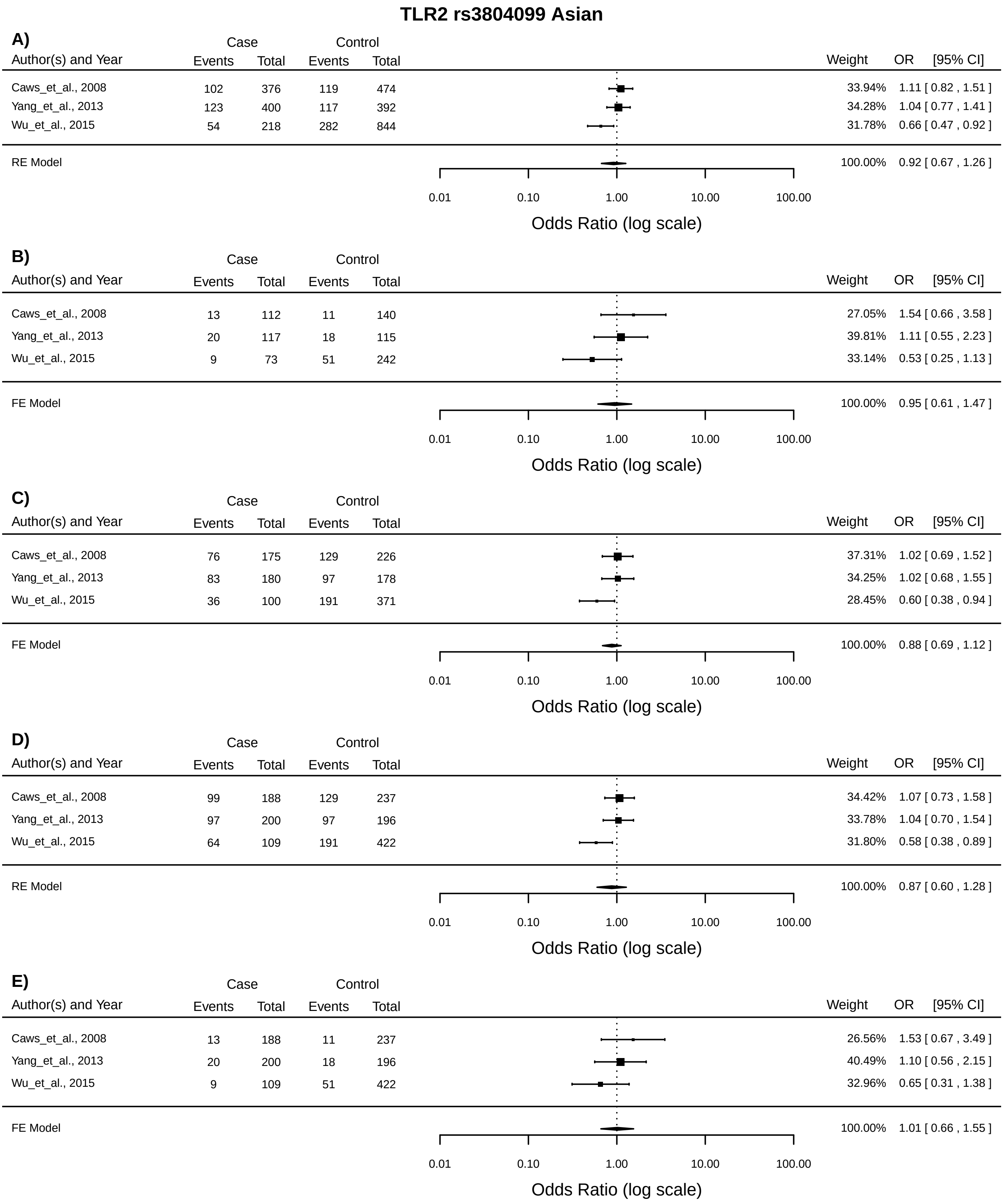
TLR2 rs3804099 Asian
A)
Author(s) and Year | Case Events | Case Total | Control Events | Control Total | Weight | OR [95% CI]
Caws_et_al., 2008 102 376 119 474 33.94% 1.11 [0.82 , 1.51]
Yang_et_al., 2013 123 400 117 392 34.28% 1.04 [0.77 , 1.41]
Wu_et_al., 2015 54 218 282 844 31.78% 0.66 [0.47 , 0.92]
RE Model 100.00% 0.92 [0.67 , 1.26]
0.01 0.10 1.00 10.00 100.00
Odds Ratio (log scale)
B)
Author(s) and Year | Case Events | Case Total | Control Events | Control Total | Weight | OR [95% CI]
Caws_et_al., 2008 13 112 11 140 27.05% 1.54 [0.66 , 3.58]
Yang_et_al., 2013 20 117 18 115 39.81% 1.11 [0.55 , 2.23]
Wu_et_al., 2015 9 73 51 242 33.14% 0.53 [0.25 , 1.13]
FE Model 100.00% 0.95 [0.61 , 1.47]
0.01 0.10 1.00 10.00 100.00
Odds Ratio (log scale)
C)
Author(s) and Year | Case Events | Case Total | Control Events | Control Total | Weight | OR [95% CI]
Caws_et_al., 2008 76 175 129 226 37.31% 1.02 [0.69 , 1.52]
Yang_et_al., 2013 83 180 97 178 34.25% 1.02 [0.68 , 1.55]
Wu_et_al., 2015 36 100 191 371 28.45% 0.60 [0.38 , 0.94]
FE Model 100.00% 0.88 [0.69 , 1.12]
0.01 0.10 1.00 10.00 100.00
Odds Ratio (log scale)
D)
Author(s) and Year | Case Events | Case Total | Control Events | Control Total | Weight | OR [95% CI]
Caws_et_al., 2008 99 188 129 237 34.42% 1.07 [0.73 , 1.58]
Yang_et_al., 2013 97 200 97 196 33.78% 1.04 [0.70 , 1.54]
Wu_et_al., 2015 64 109 191 422 31.80% 0.58 [0.38 , 0.89]
RE Model 100.00% 0.87 [0.60 , 1.28]
0.01 0.10 1.00 10.00 100.00
Odds Ratio (log scale)
E)
Author(s) and Year | Case Events | Case Total | Control Events | Control Total | Weight | OR [95% CI]
Caws_et_al., 2008 13 188 11 237 26.56% 1.53 [0.67 , 3.49]
Yang_et_al., 2013 20 200 18 196 40.49% 1.10 [0.56 , 2.15]
Wu_et_al., 2015 9 109 51 422 32.96% 0.65 [0.31 , 1.38]
FE Model 100.00% 1.01 [0.66 , 1.55]
0.01 0.10 1.00 10.00 100.00
Odds Ratio (log scale)

TLR2 rs5743708

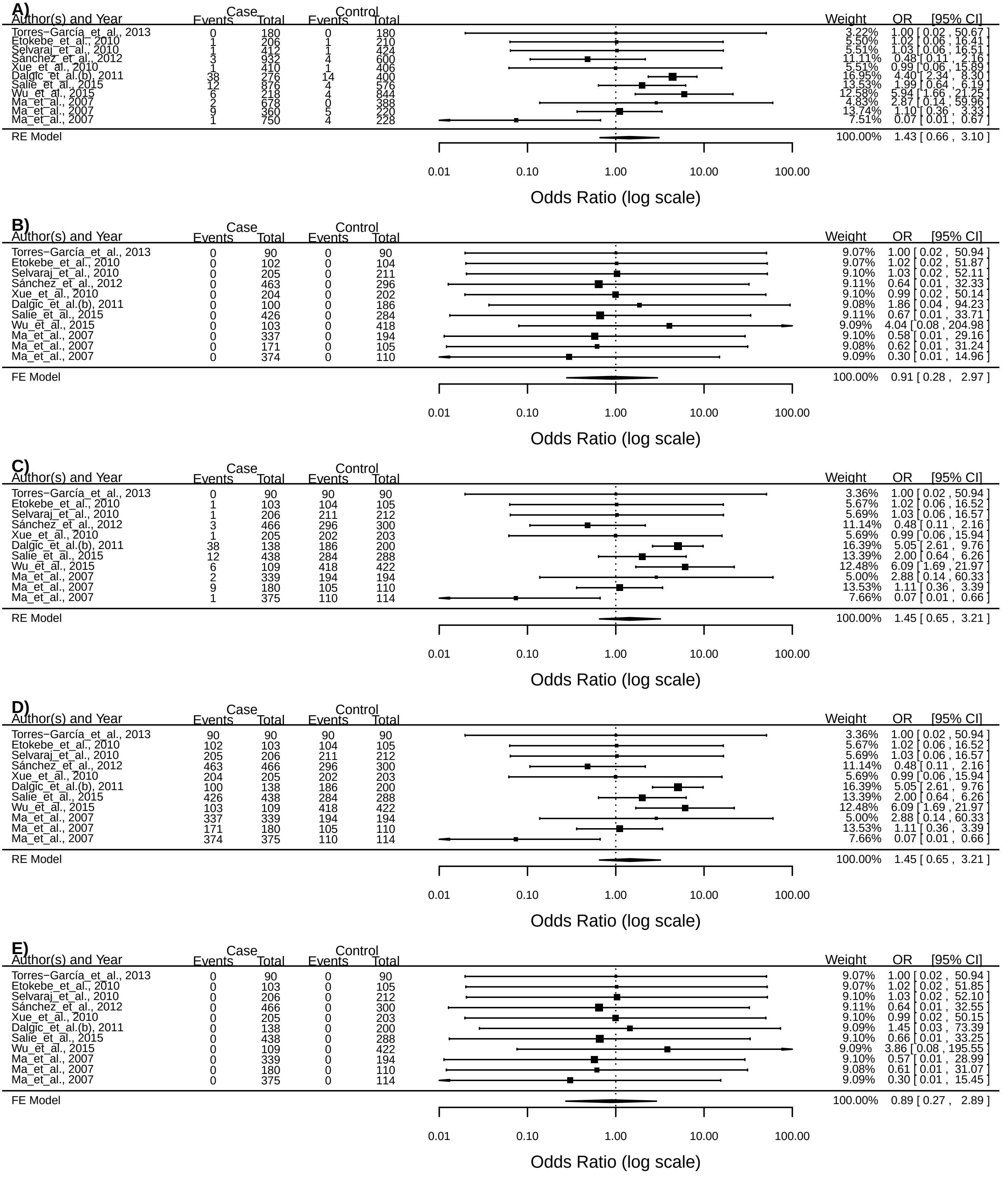

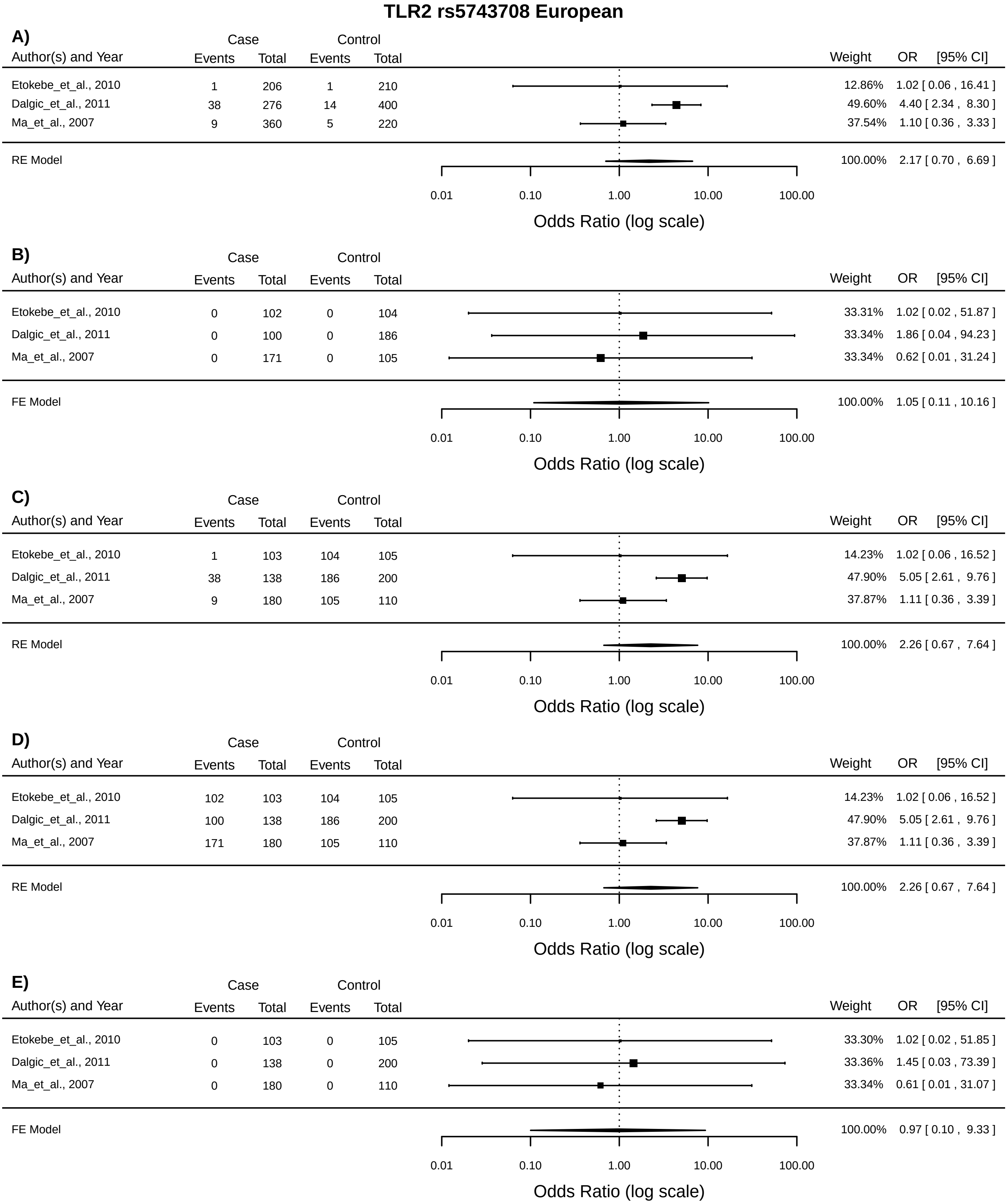
TLR2 rs5743708 European
A)
Case
Control
Author(s) and Year
Events
Total
Events
Total
Weight
OR
[95% CI]
Etokebe_et_al., 2010
1
206
1
210
12.86%
1.02 [0.06 , 16.41]
Dalgic_et_al., 2011
38
276
14
400
49.60%
4.40 [2.34 , 8.30]
Ma_et_al., 2007
9
360
5
220
37.54%
1.10 [0.36 , 3.33]
RE Model
100.00%
2.17 [0.70 , 6.69]
0.01
0.10
1.00
10.00
100.00
Odds Ratio (log scale)
B)
Case
Control
Author(s) and Year
Events
Total
Events
Total
Weight
OR
[95% CI]
Etokebe_et_al., 2010
0
102
0
104
33.31%
1.02 [0.02 , 51.87]
Dalgic_et_al., 2011
0
100
0
186
33.34%
1.86 [0.04 , 94.23]
Ma_et_al., 2007
0
171
0
105
33.34%
0.62 [0.01 , 31.24]
FE Model
100.00%
1.05 [0.11 , 10.16]
0.01
0.10
1.00
10.00
100.00
Odds Ratio (log scale)
C)
Case
Control
Author(s) and Year
Events
Total
Events
Total
Weight
OR
[95% CI]
Etokebe_et_al., 2010
1
103
104
105
14.23%
1.02 [0.06 , 16.52]
Dalgic_et_al., 2011
38
138
186
200
47.90%
5.05 [2.61 , 9.76]
Ma_et_al., 2007
9
180
105
110
37.87%
1.11 [0.36 , 3.39]
RE Model
100.00%
2.26 [0.67 , 7.64]
0.01
0.10
1.00
10.00
100.00
Odds Ratio (log scale)
D)
Case
Control
Author(s) and Year
Events
Total
Events
Total
Weight
OR
[95% CI]
Etokebe_et_al., 2010
102
103
104
105
14.23%
1.02 [0.06 , 16.52]
Dalgic_et_al., 2011
100
138
186
200
47.90%
5.05 [2.61 , 9.76]
Ma_et_al., 2007
171
180
105
110
37.87%
1.11 [0.36 , 3.39]
RE Model
100.00%
2.26 [0.67 , 7.64]
0.01
0.10
1.00
10.00
100.00
Odds Ratio (log scale)
E)
Case
Control
Author(s) and Year
Events
Total
Events
Total
Weight
OR
[95% CI]
Etokebe_et_al., 2010
0
103
0
105
33.30%
1.02 [0.02 , 51.85]
Dalgic_et_al., 2011
0
138
0
200
33.36%
1.45 [0.03 , 73.39]
Ma_et_al., 2007
0
180
0
110
33.34%
0.61 [0.01 , 31.07]
FE Model
100.00%
0.97 [0.10 , 9.33]
0.01
0.10
1.00
10.00
100.00
Odds Ratio (log scale)

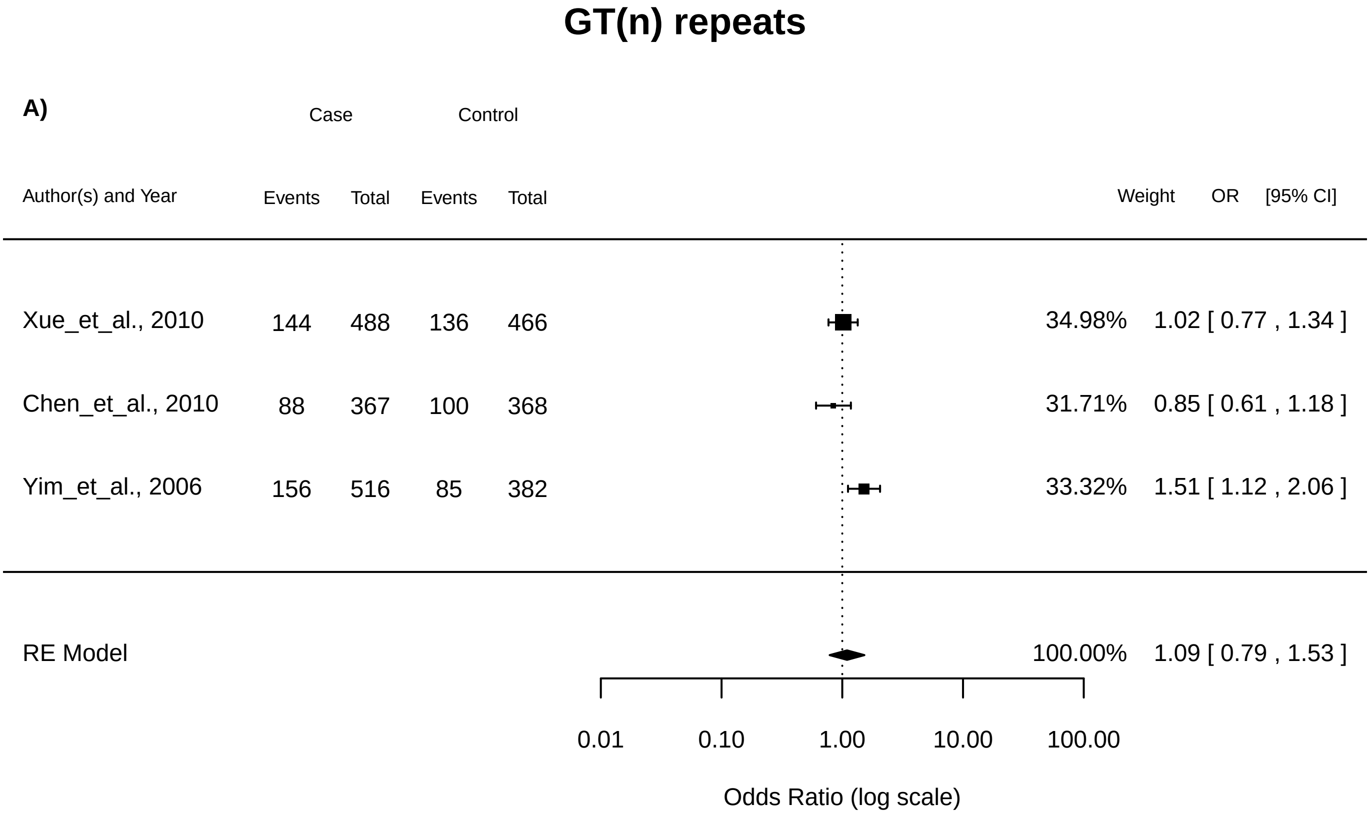
GT(n) repeats
A)
Case
Control
Author(s) and Year
Events
Total
Events
Total
Weight
OR
[95% CI]
Xue_et_al., 2010 144 488 136 466 34.98% 1.02 [0.77 , 1.34]
Chen_et_al., 2010 88 367 100 368 31.71% 0.85 [0.61 , 1.18]
Yim_et_al., 2006 156 516 85 382 33.32% 1.51 [1.12 , 2.06]
RE Model 100.00% 1.09 [0.79 , 1.53]
0.01
0.10
1.00
10.00
100.00
Odds Ratio (log scale)

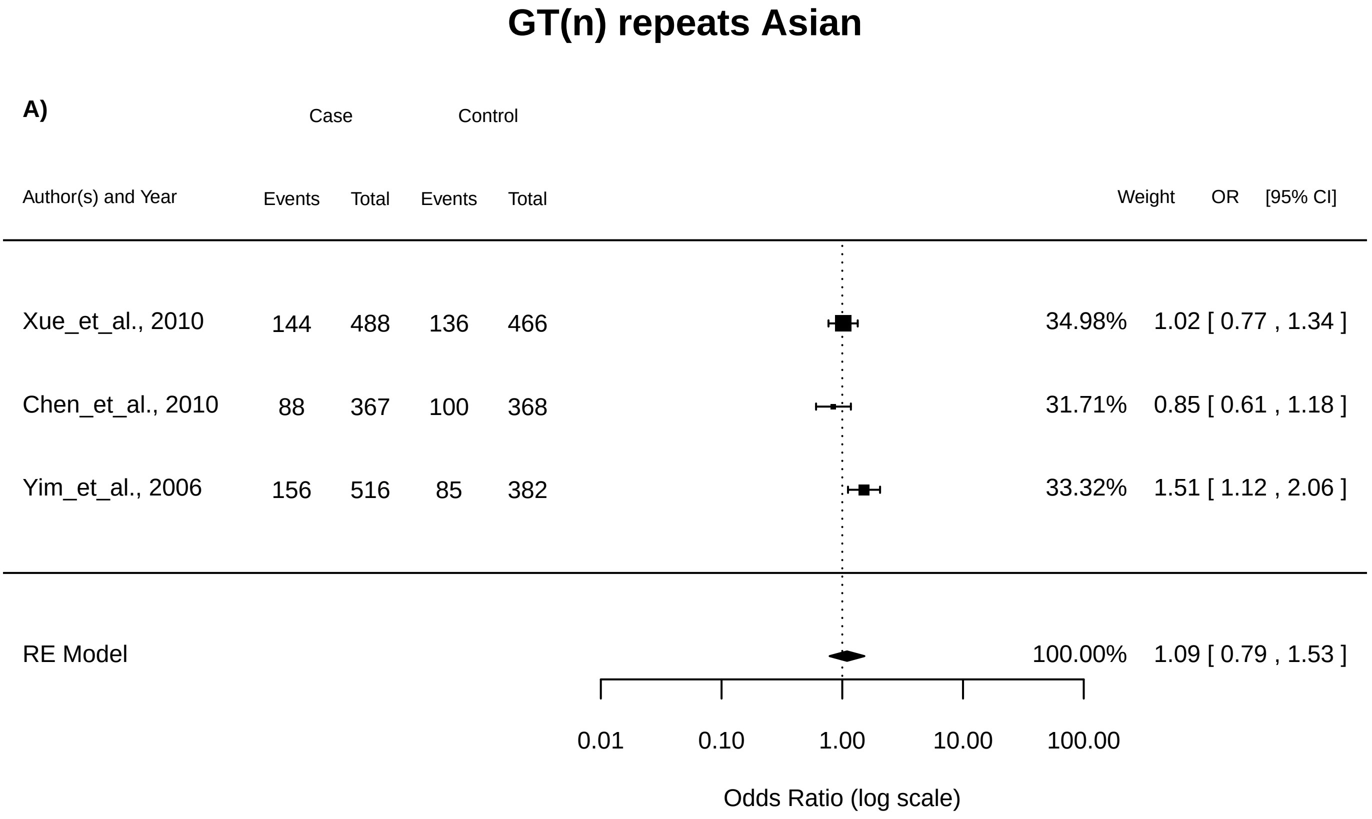
GT(n) repeats Asian
A)
Case
Control
Author(s) and Year
Events
Total
Events
Total
Weight
OR
[95% CI]
Xue_et_al., 2010 144 488 136 466 34.98% 1.02 [0.77 , 1.34]
Chen_et_al., 2010 88 367 100 368 31.71% 0.85 [0.61 , 1.18]
Yim_et_al., 2006 156 516 85 382 33.32% 1.51 [1.12 , 2.06]
RE Model 100.00% 1.09 [0.79 , 1.53]
0.01
0.10
1.00
10.00
100.00
Odds Ratio (log scale)

TLR4 rs4986790

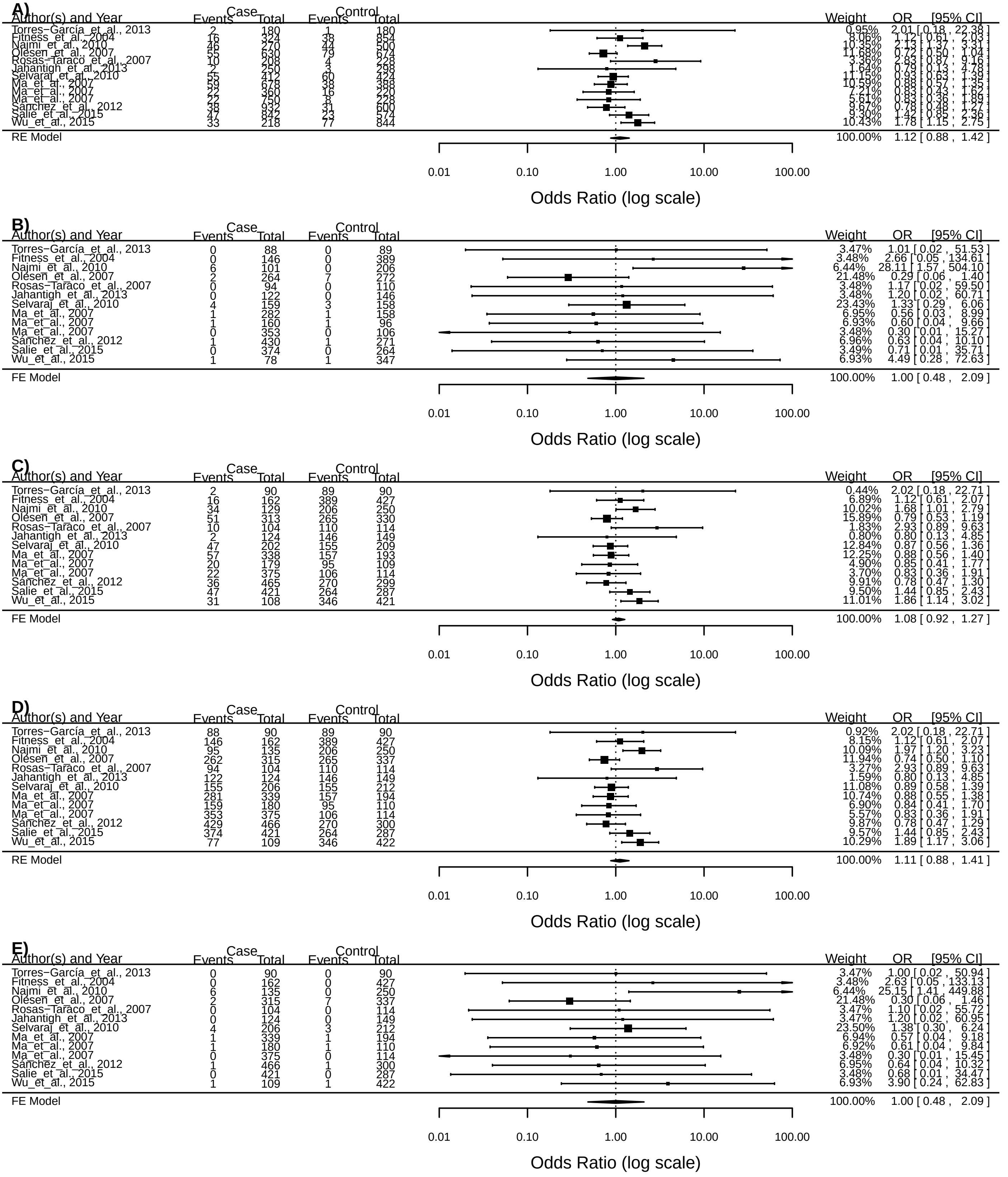

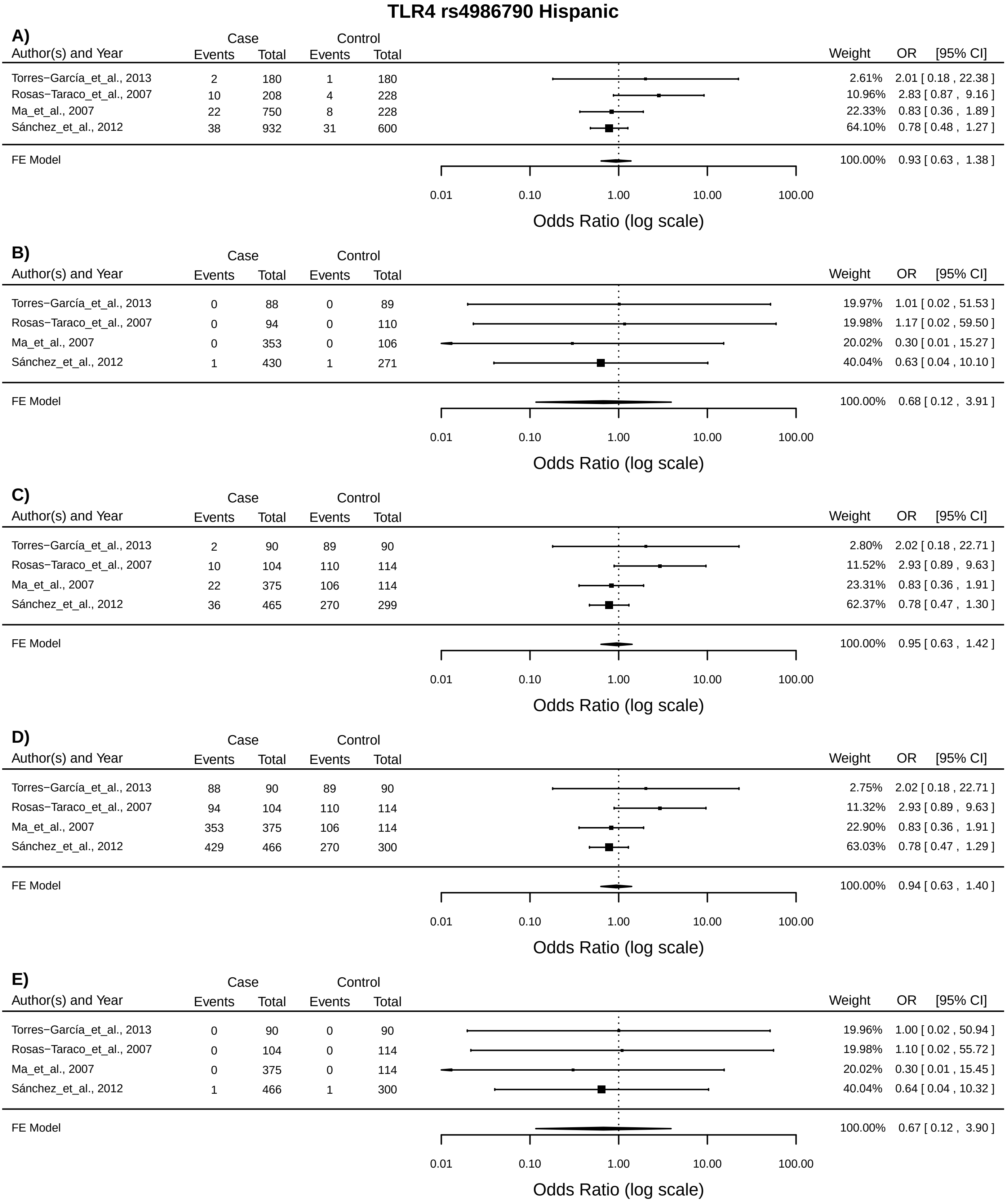
TLR4 rs4986790 Hispanic
A)
Case Control
Author(s) and Year Events Total Events Total Weight OR [95% CI]
Torres−García_et_al., 2013 2 180 1 180 2.61% 2.01 [0.18 , 22.38]
Rosas−Taraco_et_al., 2007 10 208 4 228 10.96% 2.83 [0.87 , 9.16]
Ma_et_al., 2007 22 750 8 228 22.33% 0.83 [0.36 , 1.89]
Sánchez_et_al., 2012 38 932 31 600 64.10% 0.78 [0.48 , 1.27]
FE Model 100.00% 0.93 [0.63 , 1.38]
0.01 0.10 1.00 10.00 100.00
Odds Ratio (log scale)
B)
Case Control
Author(s) and Year Events Total Events Total Weight OR [95% CI]
Torres−García_et_al., 2013 0 88 0 89 19.97% 1.01 [0.02 , 51.53]
Rosas−Taraco_et_al., 2007 0 94 0 110 19.98% 1.17 [0.02 , 59.50]
Ma_et_al., 2007 0 353 0 106 20.02% 0.30 [0.01 , 15.27]
Sánchez_et_al., 2012 1 430 1 271 40.04% 0.63 [0.04 , 10.10]
FE Model 100.00% 0.68 [0.12 , 3.91]
0.01 0.10 1.00 10.00 100.00
Odds Ratio (log scale)
C)
Case Control
Author(s) and Year Events Total Events Total Weight OR [95% CI]
Torres−García_et_al., 2013 2 90 89 90 2.80% 2.02 [0.18 , 22.71]
Rosas−Taraco_et_al., 2007 10 104 110 114 11.52% 2.93 [0.89 , 9.63]
Ma_et_al., 2007 22 375 106 114 23.31% 0.83 [0.36 , 1.91]
Sánchez_et_al., 2012 36 465 270 299 62.37% 0.78 [0.47 , 1.30]
FE Model 100.00% 0.95 [0.63 , 1.42]
0.01 0.10 1.00 10.00 100.00
Odds Ratio (log scale)
D)
Case Control
Author(s) and Year Events Total Events Total Weight OR [95% CI]
Torres−García_et_al., 2013 88 90 89 90 2.75% 2.02 [0.18 , 22.71]
Rosas−Taraco_et_al., 2007 94 104 110 114 11.32% 2.93 [0.89 , 9.63]
Ma_et_al., 2007 353 375 106 114 22.90% 0.83 [0.36 , 1.91]
Sánchez_et_al., 2012 429 466 270 300 63.03% 0.78 [0.47 , 1.29]
FE Model 100.00% 0.94 [0.63 , 1.40]
0.01 0.10 1.00 10.00 100.00
Odds Ratio (log scale)
E)
Case Control
Author(s) and Year Events Total Events Total Weight OR [95% CI]
Torres−García_et_al., 2013 0 90 0 90 19.96% 1.00 [0.02 , 50.94]
Rosas−Taraco_et_al., 2007 0 104 0 114 19.98% 1.10 [0.02 , 55.72]
Ma_et_al., 2007 0 375 0 114 20.02% 0.30 [0.01 , 15.45]
Sánchez_et_al., 2012 1 466 1 300 40.04% 0.64 [0.04 , 10.32]
FE Model 100.00% 0.67 [0.12 , 3.90]
0.01 0.10 1.00 10.00 100.00
Odds Ratio (log scale)

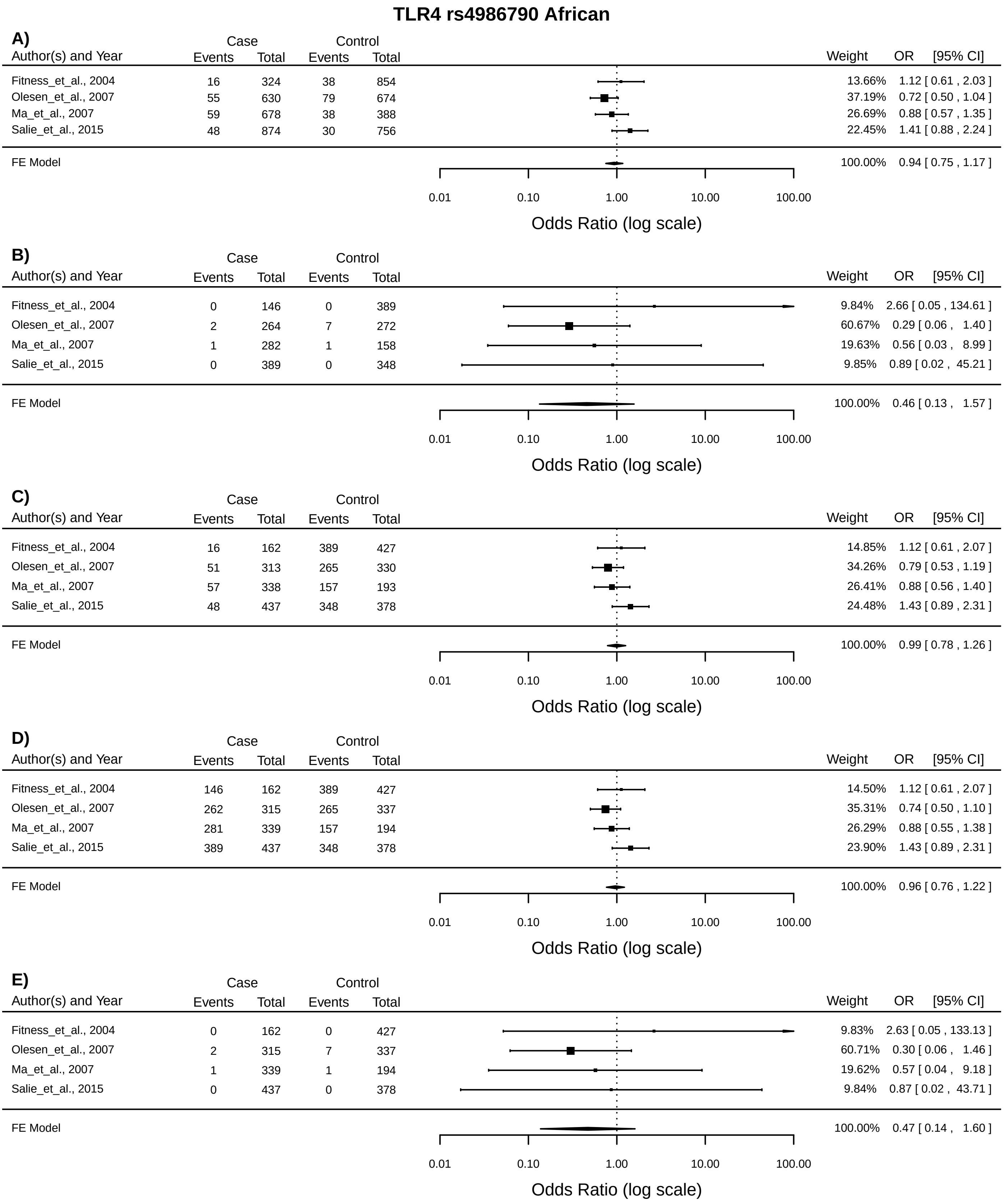
TLR4 rs4986790 African
A)
Case
Control
Author(s) and Year
Events
Total
Events
Total
Weight
OR
[95% CI]
Fitness_et_al., 2004
16
324
38
854
13.66%
1.12 [0.61 , 2.03]
Olesen_et_al., 2007
55
630
79
674
37.19%
0.72 [0.50 , 1.04]
Ma_et_al., 2007
59
678
38
388
26.69%
0.88 [0.57 , 1.35]
Salie_et_al., 2015
48
874
30
756
22.45%
1.41 [0.88 , 2.24]
FE Model
100.00%
0.94 [0.75 , 1.17]
0.01
0.10
1.00
10.00
100.00
Odds Ratio (log scale)
B)
Case
Control
Author(s) and Year
Events
Total
Events
Total
Weight
OR
[95% CI]
Fitness_et_al., 2004
0
146
0
389
9.84%
2.66 [0.05 , 134.61]
Olesen_et_al., 2007
2
264
7
272
60.67%
0.29 [0.06 , 1.40]
Ma_et_al., 2007
1
282
1
158
19.63%
0.56 [0.03 , 8.99]
Salie_et_al., 2015
0
389
0
348
9.85%
0.89 [0.02 , 45.21]
FE Model
100.00%
0.46 [0.13 , 1.57]
0.01
0.10
1.00
10.00
100.00
Odds Ratio (log scale)
C)
Case
Control
Author(s) and Year
Events
Total
Events
Total
Weight
OR
[95% CI]
Fitness_et_al., 2004
16
162
389
427
14.85%
1.12 [0.61 , 2.07]
Olesen_et_al., 2007
51
313
265
330
34.26%
0.79 [0.53 , 1.19]
Ma_et_al., 2007
57
338
157
193
26.41%
0.88 [0.56 , 1.40]
Salie_et_al., 2015
48
437
348
378
24.48%
1.43 [0.89 , 2.31]
FE Model
100.00%
0.99 [0.78 , 1.26]
0.01
0.10
1.00
10.00
100.00
Odds Ratio (log scale)
D)
Case
Control
Author(s) and Year
Events
Total
Events
Total
Weight
OR
[95% CI]
Fitness_et_al., 2004
146
162
389
427
14.50%
1.12 [0.61 , 2.07]
Olesen_et_al., 2007
262
315
265
337
35.31%
0.74 [0.50 , 1.10]
Ma_et_al., 2007
281
339
157
194
26.29%
0.88 [0.55 , 1.38]
Salie_et_al., 2015
389
437
348
378
23.90%
1.43 [0.89 , 2.31]
FE Model
100.00%
0.96 [0.76 , 1.22]
0.01
0.10
1.00
10.00
100.00
Odds Ratio (log scale)
E)
Case
Control
Author(s) and Year
Events
Total
Events
Total
Weight
OR
[95% CI]
Fitness_et_al., 2004
0
162
0
427
9.83%
2.63 [0.05 , 133.13]
Olesen_et_al., 2007
2
315
7
337
60.71%
0.30 [0.06 , 1.46]
Ma_et_al., 2007
1
339
1
194
19.62%
0.57 [0.04 , 9.18]
Salie_et_al., 2015
0
437
0
378
9.84%
0.87 [0.02 , 43.71]
FE Model
100.00%
0.47 [0.14 , 1.60]
0.01
0.10
1.00
10.00
100.00
Odds Ratio (log scale)

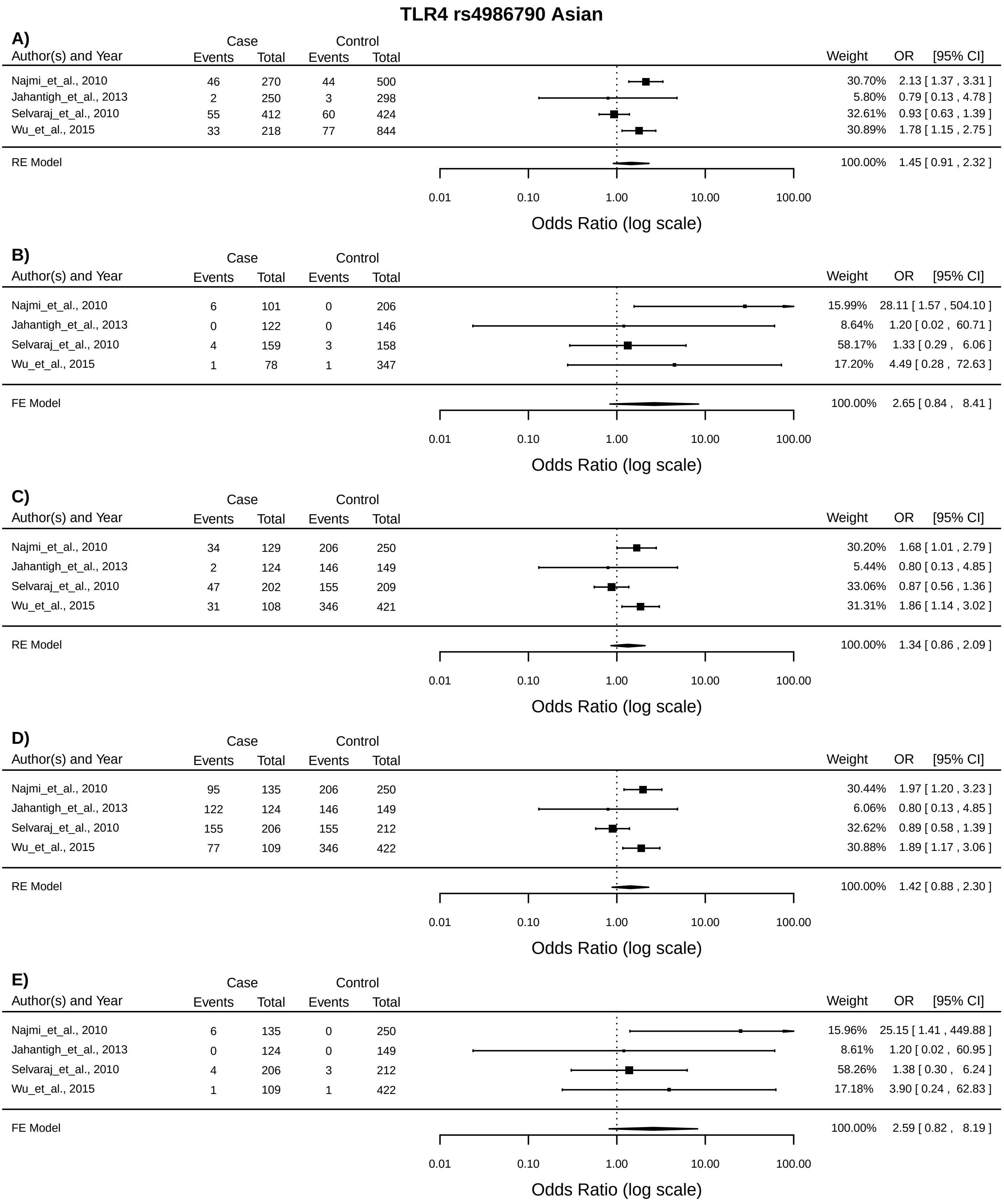
TLR4 rs4986790 Asian
A)
Case
Control
Author(s) and Year
Events Total Events Total
Weight OR [95% CI]
Najmi_et_al., 2010 46 270 44 500 30.70% 2.13 [1.37 , 3.31]
Jahantigh_et_al., 2013 2 250 3 298 5.80% 0.79 [0.13 , 4.78]
Selvaraj_et_al., 2010 55 412 60 424 32.61% 0.93 [0.63 , 1.39]
Wu_et_al., 2015 33 218 77 844 30.89% 1.78 [1.15 , 2.75]
RE Model 100.00% 1.45 [0.91 , 2.32]
0.01 0.10 1.00 10.00 100.00
Odds Ratio (log scale)
B)
Case
Control
Author(s) and Year
Events Total Events Total
Weight OR [95% CI]
Najmi_et_al., 2010 6 101 0 206 15.99% 28.11 [1.57 , 504.10]
Jahantigh_et_al., 2013 0 122 0 146 8.64% 1.20 [0.02 , 60.71]
Selvaraj_et_al., 2010 4 159 3 158 58.17% 1.33 [0.29 , 6.06]
Wu_et_al., 2015 1 78 1 347 17.20% 4.49 [0.28 , 72.63]
FE Model 100.00% 2.65 [0.84 , 8.41]
0.01 0.10 1.00 10.00 100.00
Odds Ratio (log scale)
C)
Case
Control
Author(s) and Year
Events Total Events Total
Weight OR [95% CI]
Najmi_et_al., 2010 34 129 206 250 30.20% 1.68 [1.01 , 2.79]
Jahantigh_et_al., 2013 2 124 146 149 5.44% 0.80 [0.13 , 4.85]
Selvaraj_et_al., 2010 47 202 155 209 33.06% 0.87 [0.56 , 1.36]
Wu_et_al., 2015 31 108 346 421 31.31% 1.86 [1.14 , 3.02]
RE Model 100.00% 1.34 [0.86 , 2.09]
0.01 0.10 1.00 10.00 100.00
Odds Ratio (log scale)
D)
Case
Control
Author(s) and Year
Events Total Events Total
Weight OR [95% CI]
Najmi_et_al., 2010 95 135 206 250 30.44% 1.97 [1.20 , 3.23]
Jahantigh_et_al., 2013 122 124 146 149 6.06% 0.80 [0.13 , 4.85]
Selvaraj_et_al., 2010 155 206 155 212 32.62% 0.89 [0.58 , 1.39]
Wu_et_al., 2015 77 109 346 422 30.88% 1.89 [1.17 , 3.06]
RE Model 100.00% 1.42 [0.88 , 2.30]
0.01 0.10 1.00 10.00 100.00
Odds Ratio (log scale)
E)
Case
Control
Author(s) and Year
Events Total Events Total
Weight OR [95% CI]
Najmi_et_al., 2010 6 135 0 250 15.96% 25.15 [1.41 , 449.88]
Jahantigh_et_al., 2013 0 124 0 149 8.61% 1.20 [0.02 , 60.95]
Selvaraj_et_al., 2010 4 206 3 212 58.26% 1.38 [0.30 , 6.24]
Wu_et_al., 2015 1 109 1 422 17.18% 3.90 [0.24 , 62.83]
FE Model 100.00% 2.59 [0.82 , 8.19]
0.01 0.10 1.00 10.00 100.00
Odds Ratio (log scale)

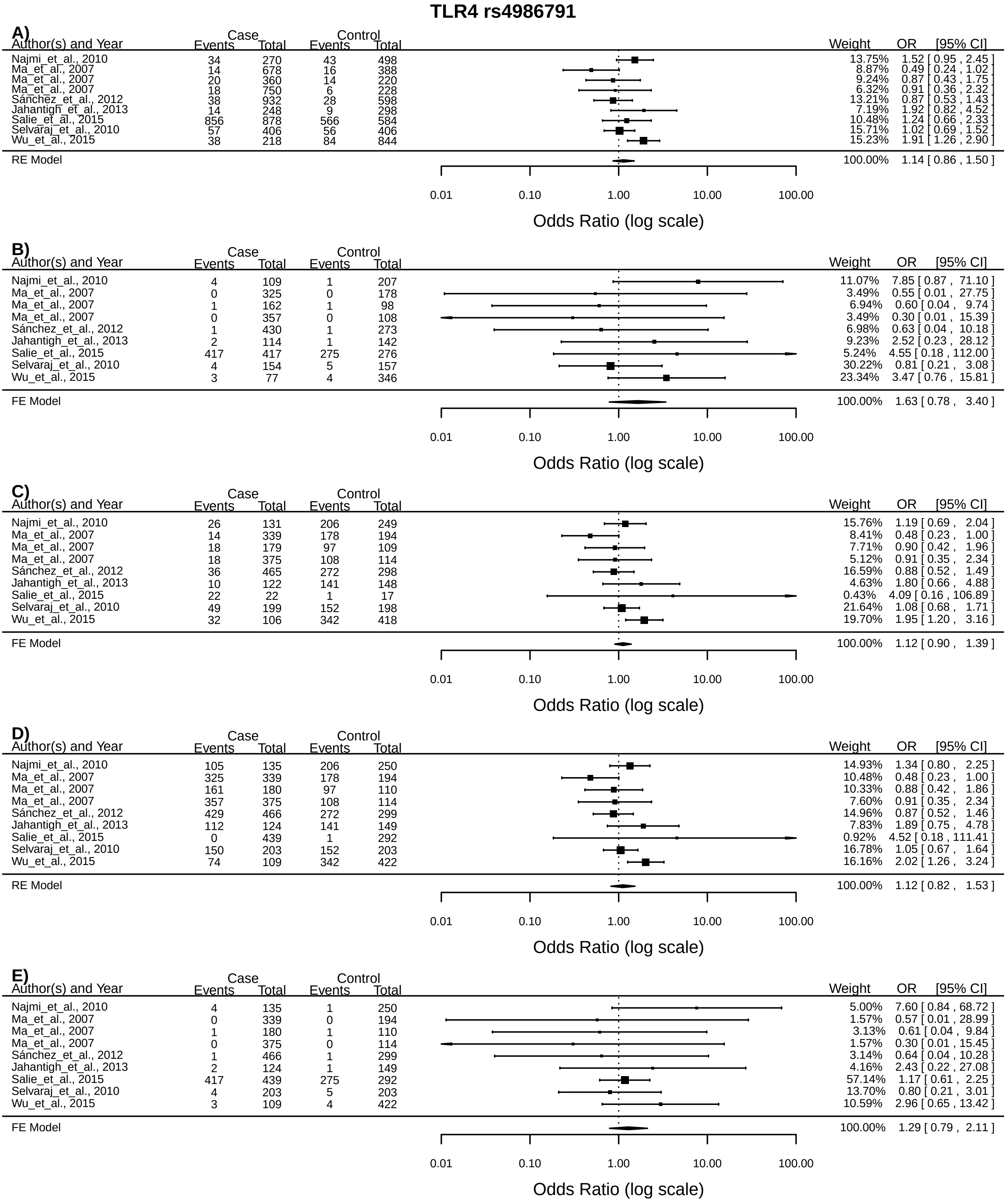
TLR4 rs4986791
A)
Author(s) and Year | Case Events | Case Total | Control Events | Control Total | Weight | OR [95% CI]
Najmi_et_al., 2010 | 34 | 270 | 43 | 498 | 13.75% | 1.52 [0.95 , 2.45]
Ma_et_al., 2007 | 14 | 678 | 16 | 388 | 8.87% | 0.49 [0.24 , 1.02]
Ma_et_al., 2007 | 20 | 360 | 14 | 220 | 9.24% | 0.87 [0.43 , 1.75]
Ma_et_al., 2007 | 18 | 750 | 6 | 228 | 6.32% | 0.91 [0.36 , 2.32]
Sánchez_et_al., 2012 | 38 | 932 | 28 | 598 | 13.21% | 0.87 [0.53 , 1.43]
Jahantigh_et_al., 2013 | 14 | 248 | 9 | 298 | 7.19% | 1.92 [0.82 , 4.52]
Salie_et_al., 2015 | 856 | 878 | 566 | 584 | 10.48% | 1.24 [0.66 , 2.33]
Selvaraj_et_al., 2010 | 57 | 406 | 56 | 406 | 15.71% | 1.02 [0.69 , 1.52]
Wu_et_al., 2015 | 38 | 218 | 84 | 844 | 15.23% | 1.91 [1.26 , 2.90]
RE Model | 100.00% | 1.14 [0.86 , 1.50]
0.01 0.10 1.00 10.00 100.00
Odds Ratio (log scale)
B)
Author(s) and Year | Case Events | Case Total | Control Events | Control Total | Weight | OR [95% CI]
Najmi_et_al., 2010 | 4 | 109 | 1 | 207 | 11.07% | 7.85 [0.87 , 71.10]
Ma_et_al., 2007 | 0 | 325 | 0 | 178 | 3.49% | 0.55 [0.01 , 27.75]
Ma_et_al., 2007 | 1 | 162 | 1 | 98 | 6.94% | 0.60 [0.04 , 9.74]
Ma_et_al., 2007 | 0 | 357 | 0 | 108 | 3.49% | 0.30 [0.01 , 15.39]
Sánchez_et_al., 2012 | 1 | 430 | 1 | 273 | 6.98% | 0.63 [0.04 , 10.18]
Jahantigh_et_al., 2013 | 2 | 114 | 1 | 142 | 9.23% | 2.52 [0.23 , 28.12]
Salie_et_al., 2015 | 417 | 417 | 275 | 276 | 5.24% | 4.55 [0.18 , 112.00]
Selvaraj_et_al., 2010 | 4 | 154 | 5 | 157 | 30.22% | 0.81 [0.21 , 3.08]
Wu_et_al., 2015 | 3 | 77 | 4 | 346 | 23.34% | 3.47 [0.76 , 15.81]
FE Model | 100.00% | 1.63 [0.78 , 3.40]
0.01 0.10 1.00 10.00 100.00
Odds Ratio (log scale)
C)
Author(s) and Year | Case Events | Case Total | Control Events | Control Total | Weight | OR [95% CI]
Najmi_et_al., 2010 | 26 | 131 | 206 | 249 | 15.76% | 1.19 [0.69 , 2.04]
Ma_et_al., 2007 | 14 | 339 | 178 | 194 | 8.41% | 0.48 [0.23 , 1.00]
Ma_et_al., 2007 | 18 | 179 | 97 | 109 | 7.71% | 0.90 [0.42 , 1.96]
Ma_et_al., 2007 | 18 | 375 | 108 | 114 | 5.12% | 0.91 [0.35 , 2.34]
Sánchez_et_al., 2012 | 36 | 465 | 272 | 298 | 16.59% | 0.88 [0.52 , 1.49]
Jahantigh_et_al., 2013 | 10 | 122 | 141 | 148 | 4.63% | 1.80 [0.66 , 4.88]
Salie_et_al., 2015 | 22 | 22 | 1 | 17 | 0.43% | 4.09 [0.16 , 106.89]
Selvaraj_et_al., 2010 | 49 | 199 | 152 | 198 | 21.64% | 1.08 [0.68 , 1.71]
Wu_et_al., 2015 | 32 | 106 | 342 | 418 | 19.70% | 1.95 [1.20 , 3.16]
FE Model | 100.00% | 1.12 [0.90 , 1.39]
0.01 0.10 1.00 10.00 100.00
Odds Ratio (log scale)
D)
Author(s) and Year | Case Events | Case Total | Control Events | Control Total | Weight | OR [95% CI]
Najmi_et_al., 2010 | 105 | 135 | 206 | 250 | 14.93% | 1.34 [0.80 , 2.25]
Ma_et_al., 2007 | 325 | 339 | 178 | 194 | 10.48% | 0.48 [0.23 , 1.00]
Ma_et_al., 2007 | 161 | 180 | 97 | 110 | 10.33% | 0.88 [0.42 , 1.86]
Ma_et_al., 2007 | 357 | 375 | 108 | 114 | 7.60% | 0.91 [0.35 , 2.34]
Sánchez_et_al., 2012 | 429 | 466 | 272 | 299 | 14.96% | 0.87 [0.52 , 1.46]
Jahantigh_et_al., 2013 | 112 | 124 | 141 | 149 | 7.83% | 1.89 [0.75 , 4.78]
Salie_et_al., 2015 | 0 | 439 | 1 | 292 | 0.92% | 4.52 [0.18 , 111.41]
Selvaraj_et_al., 2010 | 150 | 203 | 152 | 203 | 16.78% | 1.05 [0.67 , 1.64]
Wu_et_al., 2015 | 74 | 109 | 342 | 422 | 16.16% | 2.02 [1.26 , 3.24]
RE Model | 100.00% | 1.12 [0.82 , 1.53]
0.01 0.10 1.00 10.00 100.00
Odds Ratio (log scale)
E)
Author(s) and Year | Case Events | Case Total | Control Events | Control Total | Weight | OR [95% CI]
Najmi_et_al., 2010 | 4 | 135 | 1 | 250 | 5.00% | 7.60 [0.84 , 68.72]
Ma_et_al., 2007 | 0 | 339 | 0 | 194 | 1.57% | 0.57 [0.01 , 28.99]
Ma_et_al., 2007 | 1 | 180 | 1 | 110 | 3.13% | 0.61 [0.04 , 9.84]
Ma_et_al., 2007 | 0 | 375 | 0 | 114 | 1.57% | 0.30 [0.01 , 15.45]
Sánchez_et_al., 2012 | 1 | 466 | 1 | 299 | 3.14% | 0.64 [0.04 , 10.28]
Jahantigh_et_al., 2013 | 2 | 124 | 1 | 149 | 4.16% | 2.43 [0.22 , 27.08]
Salie_et_al., 2015 | 417 | 439 | 275 | 292 | 57.14% | 1.17 [0.61 , 2.25]
Selvaraj_et_al., 2010 | 4 | 203 | 5 | 203 | 13.70% | 0.80 [0.21 , 3.01]
Wu_et_al., 2015 | 3 | 109 | 4 | 422 | 10.59% | 2.96 [0.65 , 13.42]
FE Model | 100.00% | 1.29 [0.79 , 2.11]
0.01 0.10 1.00 10.00 100.00
Odds Ratio (log scale)

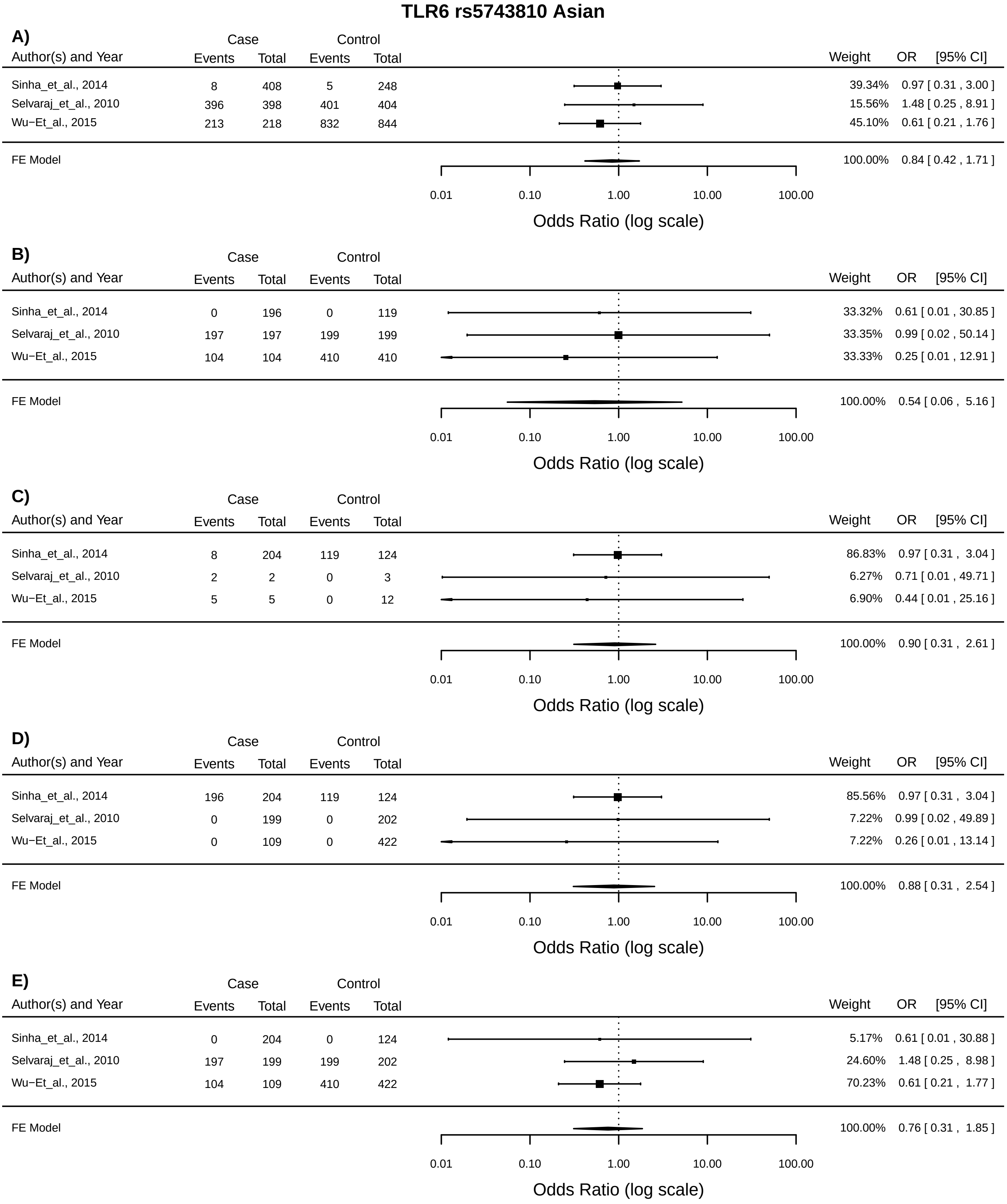
TLR6 rs5743810 Asian
A)
Case
Control
Author(s) and Year
Events
Total
Events
Total
Weight
OR
[95% CI]
Sinha_et_al., 2014
8
408
5
248
39.34%
0.97 [0.31 , 3.00]
Selvaraj_et_al., 2010
396
398
401
404
15.56%
1.48 [0.25 , 8.91]
Wu−Et_al., 2015
213
218
832
844
45.10%
0.61 [0.21 , 1.76]
FE Model
100.00%
0.84 [0.42 , 1.71]
0.01
0.10
1.00
10.00
100.00
Odds Ratio (log scale)
B)
Case
Control
Author(s) and Year
Events
Total
Events
Total
Weight
OR
[95% CI]
Sinha_et_al., 2014
0
196
0
119
33.32%
0.61 [0.01 , 30.85]
Selvaraj_et_al., 2010
197
197
199
199
33.35%
0.99 [0.02 , 50.14]
Wu−Et_al., 2015
104
104
410
410
33.33%
0.25 [0.01 , 12.91]
FE Model
100.00%
0.54 [0.06 , 5.16]
0.01
0.10
1.00
10.00
100.00
Odds Ratio (log scale)
C)
Case
Control
Author(s) and Year
Events
Total
Events
Total
Weight
OR
[95% CI]
Sinha_et_al., 2014
8
204
119
124
86.83%
0.97 [0.31 , 3.04]
Selvaraj_et_al., 2010
2
2
0
3
6.27%
0.71 [0.01 , 49.71]
Wu−Et_al., 2015
5
5
0
12
6.90%
0.44 [0.01 , 25.16]
FE Model
100.00%
0.90 [0.31 , 2.61]
0.01
0.10
1.00
10.00
100.00
Odds Ratio (log scale)
D)
Case
Control
Author(s) and Year
Events
Total
Events
Total
Weight
OR
[95% CI]
Sinha_et_al., 2014
196
204
119
124
85.56%
0.97 [0.31 , 3.04]
Selvaraj_et_al., 2010
0
199
0
202
7.22%
0.99 [0.02 , 49.89]
Wu−Et_al., 2015
0
109
0
422
7.22%
0.26 [0.01 , 13.14]
FE Model
100.00%
0.88 [0.31 , 2.54]
0.01
0.10
1.00
10.00
100.00
Odds Ratio (log scale)
E)
Case
Control
Author(s) and Year
Events
Total
Events
Total
Weight
OR
[95% CI]
Sinha_et_al., 2014
0
204
0
124
5.17%
0.61 [0.01 , 30.88]
Selvaraj_et_al., 2010
197
199
199
202
24.60%
1.48 [0.25 , 8.98]
Wu−Et_al., 2015
104
109
410
422
70.23%
0.61 [0.21 , 1.77]
FE Model
100.00%
0.76 [0.31 , 1.85]
0.01
0.10
1.00
10.00
100.00
Odds Ratio (log scale)

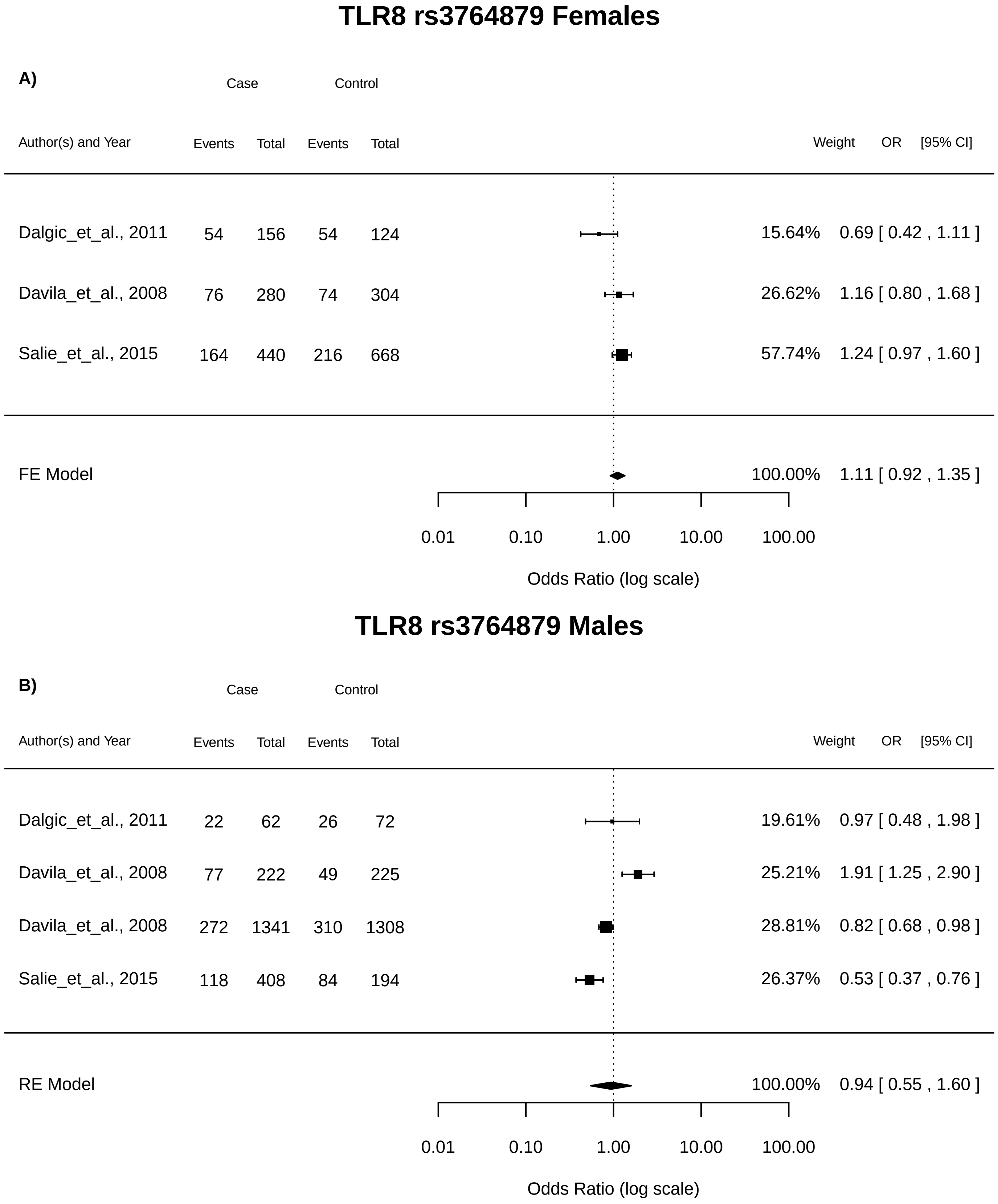
TLR8 rs3764879 Females
A)
Case
Control
Author(s) and Year
Events
Total
Events
Total
Weight
OR
[95% CI]
Dalgic_et_al., 2011 54 156 54 124 15.64% 0.69 [0.42 , 1.11]
Davila_et_al., 2008 76 280 74 304 26.62% 1.16 [0.80 , 1.68]
Salie_et_al., 2015 164 440 216 668 57.74% 1.24 [0.97 , 1.60]
FE Model 100.00% 1.11 [0.92 , 1.35]
0.01
0.10
1.00
10.00
100.00
Odds Ratio (log scale)
TLR8 rs3764879 Males
B)
Case
Control
Author(s) and Year
Events
Total
Events
Total
Weight
OR
[95% CI]
Dalgic_et_al., 2011 22 62 26 72 19.61% 0.97 [0.48 , 1.98]
Davila_et_al., 2008 77 222 49 225 25.21% 1.91 [1.25 , 2.90]
Davila_et_al., 2008 272 1341 310 1308 28.81% 0.82 [0.68 , 0.98]
Salie_et_al., 2015 118 408 84 194 26.37% 0.53 [0.37 , 0.76]
RE Model 100.00% 0.94 [0.55 , 1.60]
0.01
0.10
1.00
10.00
100.00
Odds Ratio (log scale)

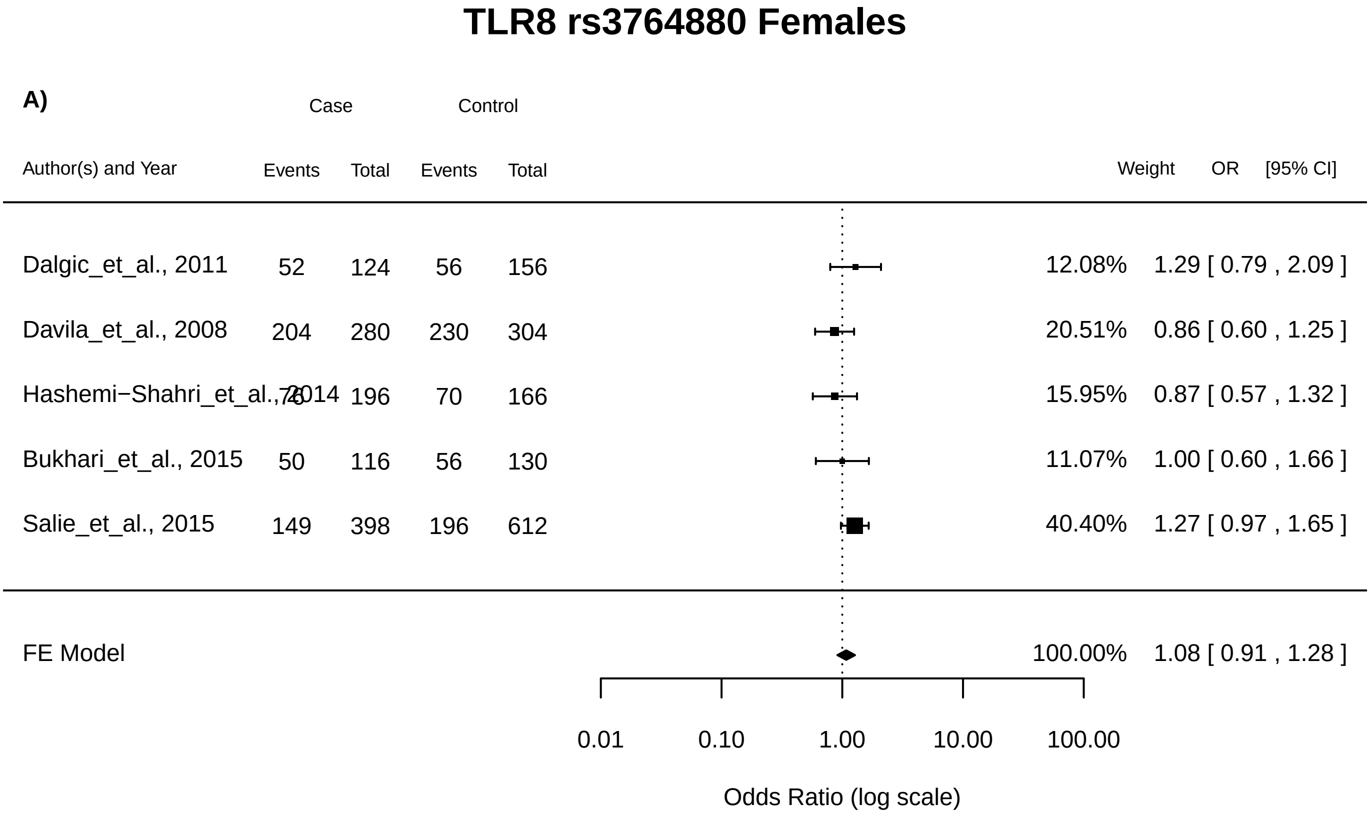
TLR8 rs3764880 Females
A)
Case
Control
Author(s) and Year
Events
Total
Events
Total
Weight
OR
[95% CI]
Dalgic_et_al., 2011 52 124 56 156 12.08% 1.29 [0.79 , 2.09]
Davila_et_al., 2008 204 280 230 304 20.51% 0.86 [0.60 , 1.25]
Hashemi−Shahri_et_al., 2014 76 196 70 166 15.95% 0.87 [0.57 , 1.32]
Bukhari_et_al., 2015 50 116 56 130 11.07% 1.00 [0.60 , 1.66]
Salie_et_al., 2015 149 398 196 612 40.40% 1.27 [0.97 , 1.65]
FE Model 100.00% 1.08 [0.91 , 1.28]
0.01
0.10
1.00
10.00
100.00
Odds Ratio (log scale)

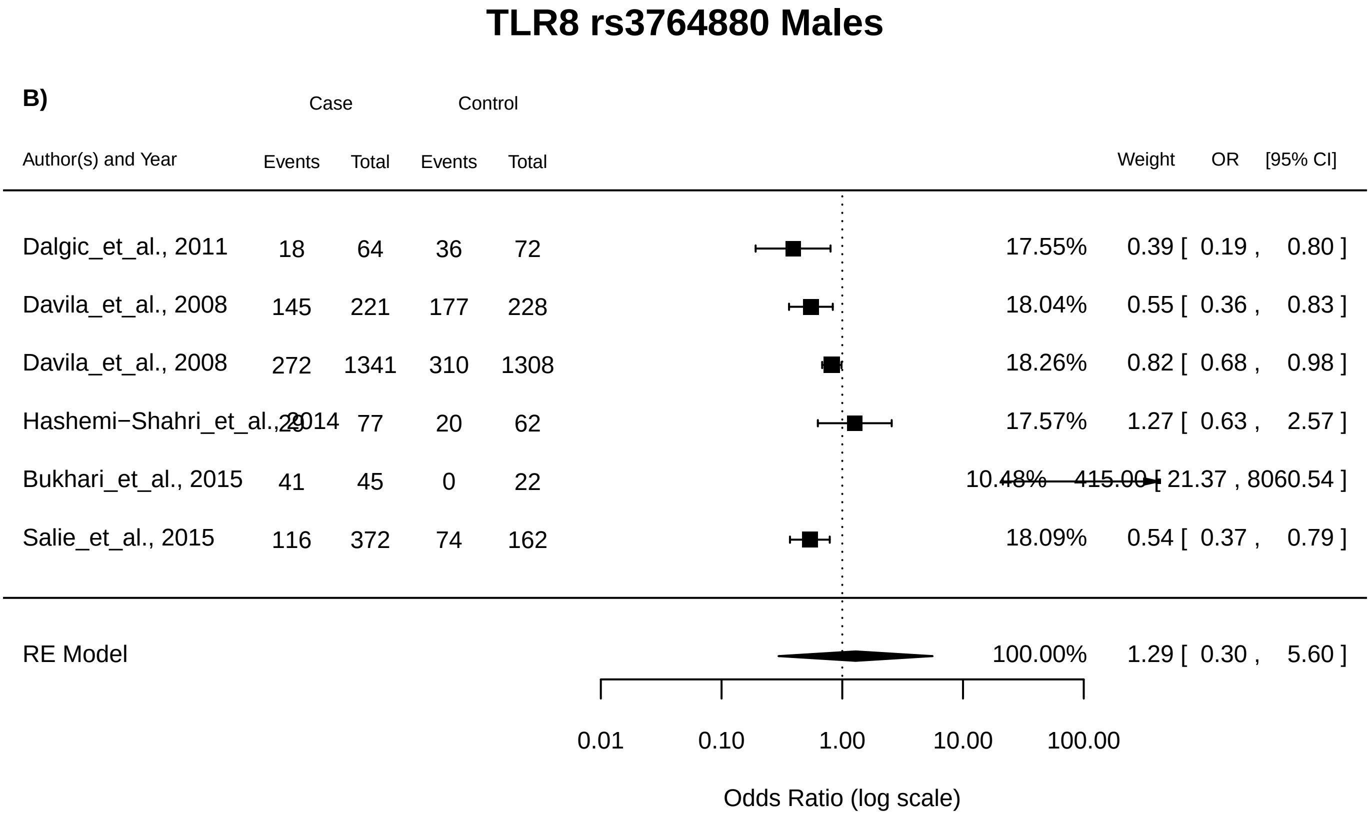
TLR8 rs3764880 Males
B)
Case
Control
Author(s) and Year
Events
Total
Events
Total
Weight
OR
[95% CI]
Dalgic_et_al., 2011 18 64 36 72 17.55% 0.39 [0.19 , 0.80]
Davila_et_al., 2008 145 221 177 228 18.04% 0.55 [0.36 , 0.83]
Davila_et_al., 2008 272 1341 310 1308 18.26% 0.82 [0.68 , 0.98]
Hashemi−Shahri_et_al., 2014 29 77 20 62 17.57% 1.27 [0.63 , 2.57]
Bukhari_et_al., 2015 41 45 0 22 10.48% 415.00 [21.37 , 8060.54]
Salie_et_al., 2015 116 372 74 162 18.09% 0.54 [0.37 , 0.79]
RE Model 100.00% 1.29 [0.30 , 5.60]
0.01
0.10
1.00
10.00
100.00
Odds Ratio (log scale)

TLR9 rs5743836

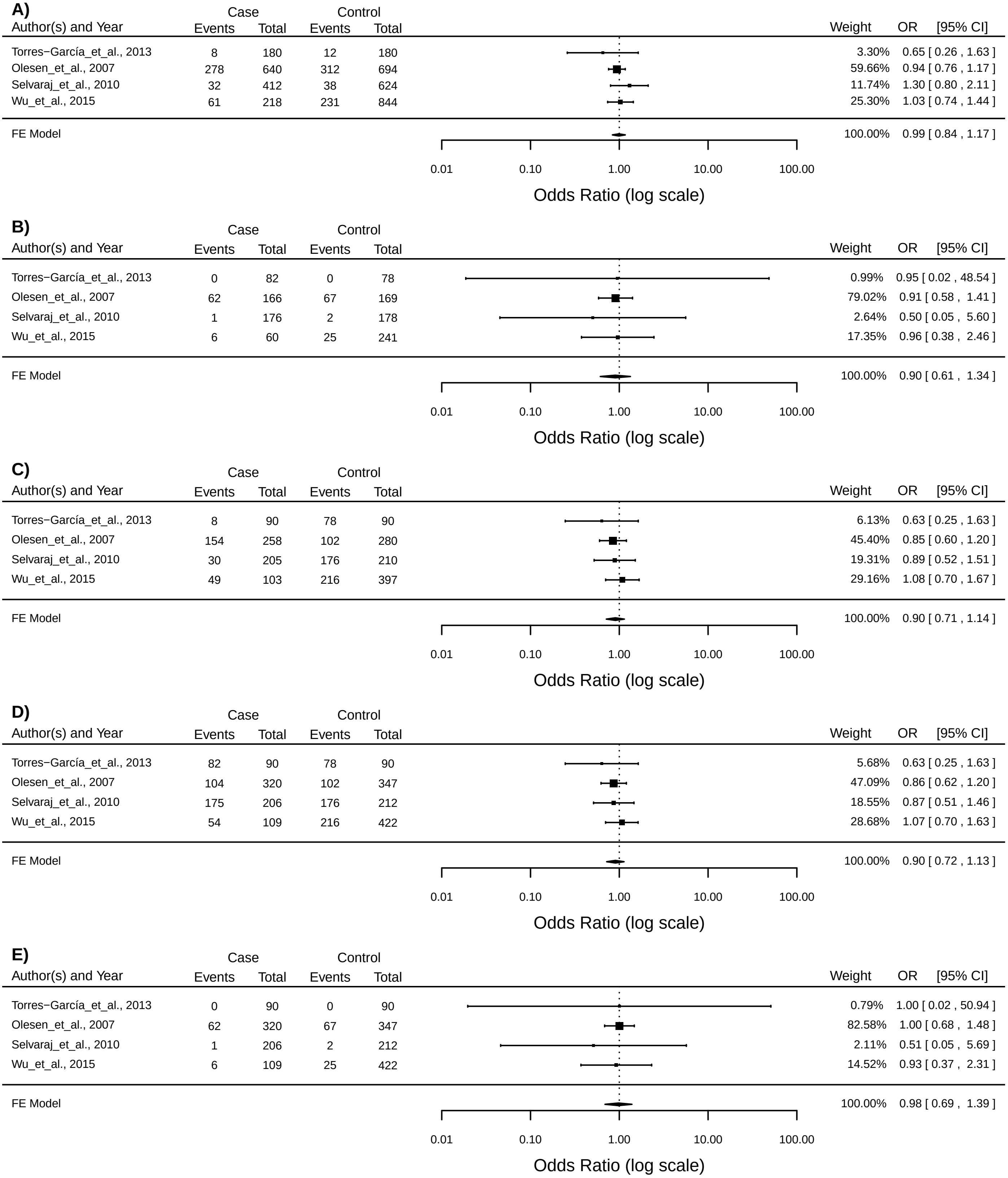

TLR9 rs1870884

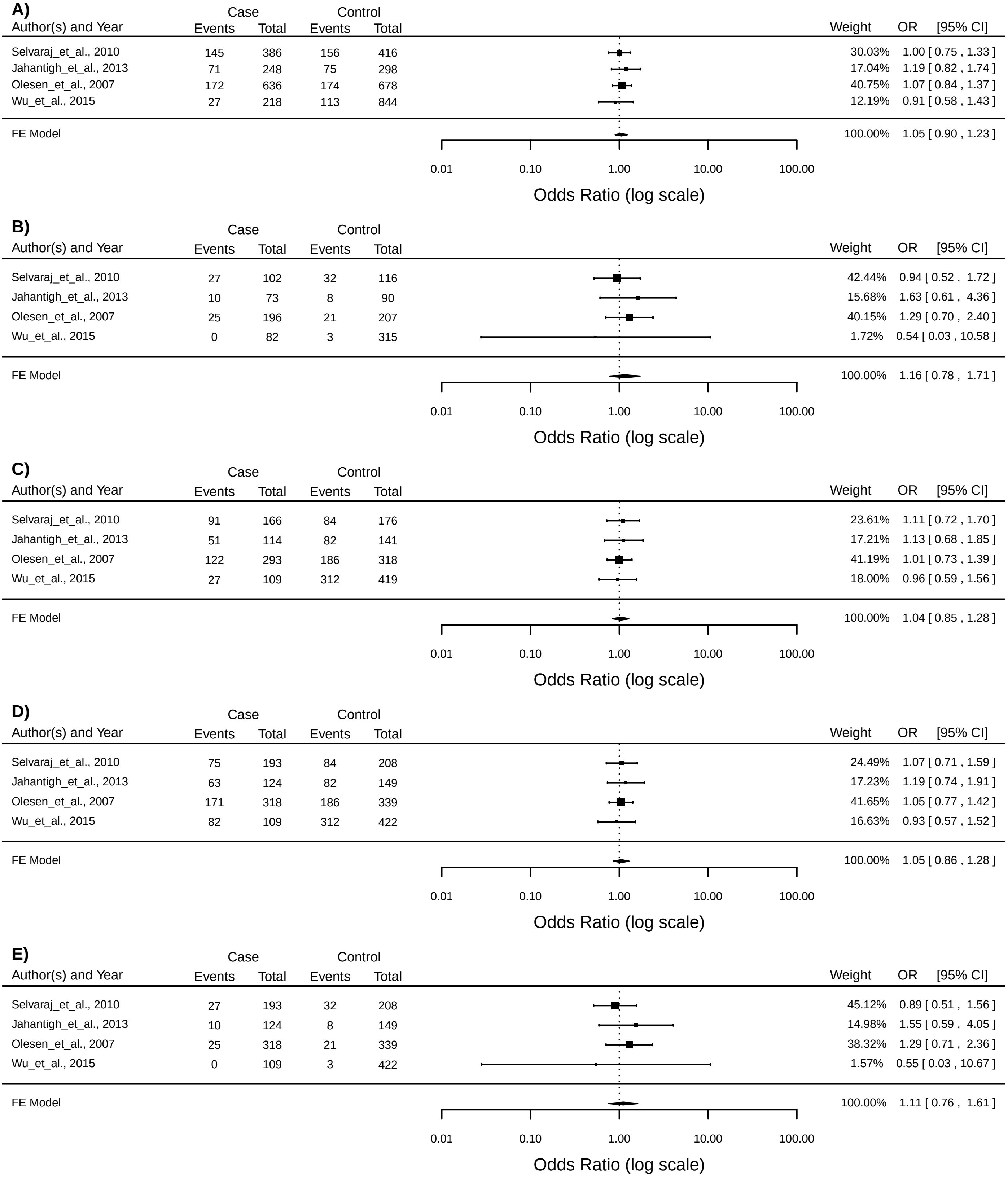

TLR9 rs1870884 Asian

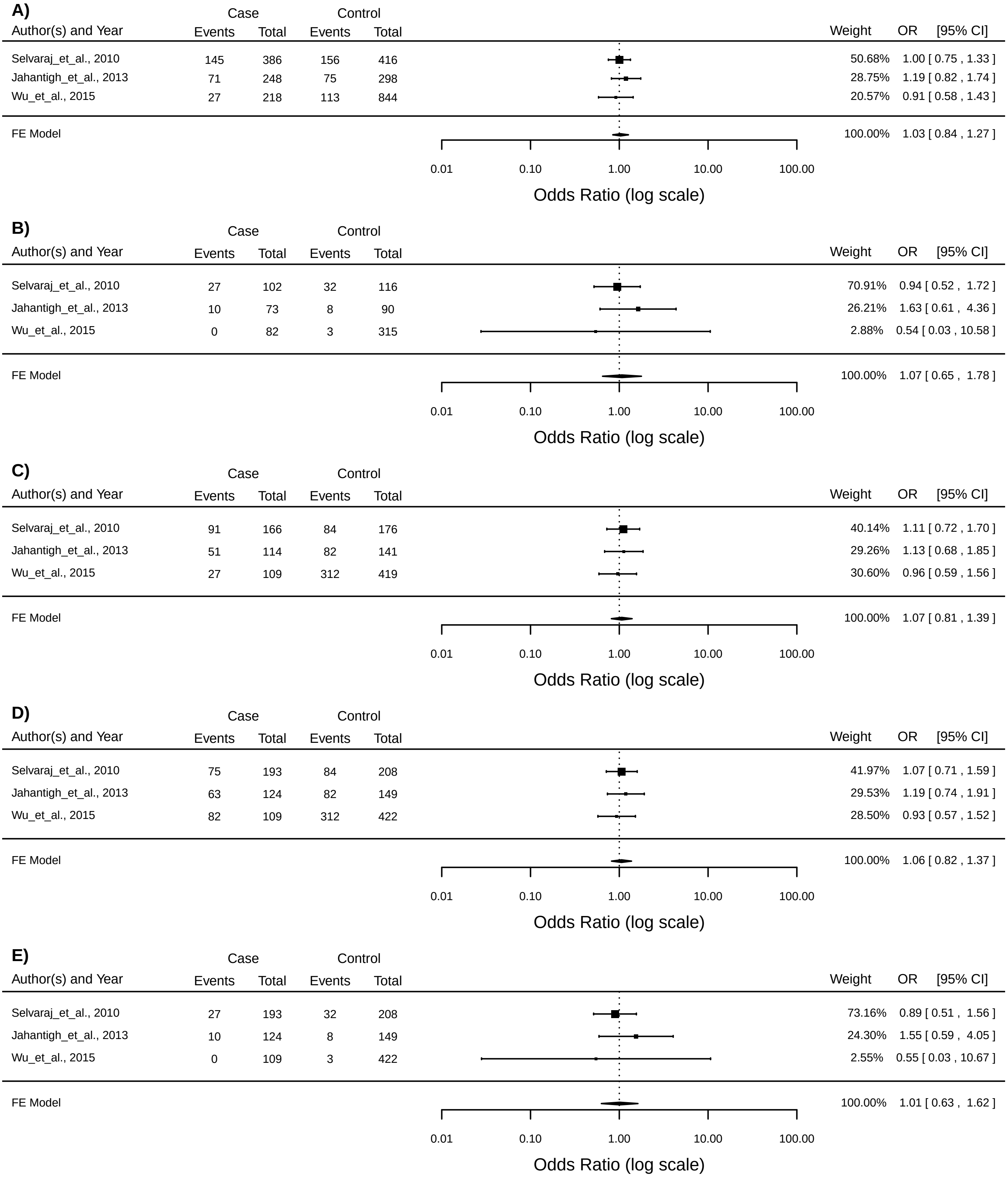

Supplement: S1 Fig — A) Allelic model, Ai) Allelic model following D&T correction, B) Homozygote comparison, C) Heterozygote comparison, Ci) Heterozygote comparison following D&T correction, D) Dominant model, Di) Dominant model following D&T correction, E) Recessive model. OR: odds ratio; 95%CI: 95% confidence interval; D&T: Duval and Tweedie. (PDF) [file pone.0139711.s001.pdf]
